# A novel model of central precocious puberty disease: Paternal *MKRN3* gene–modified rabbit

**DOI:** 10.1002/ame2.12544

**Published:** 2025-01-24

**Authors:** Bangzhu Chen, Xing Ye, Lihao Chen, Tianping Liu, Guiling Li, Chula Sa, Juan Li, Ke Liu, Weiwang Gu, Gang Wang

**Affiliations:** ^1^ Department of Obstetrics and Gynecology, Guangdong Provincial Key Laboratory of Major Obstetric Diseases, Guangdong Provincial Clinical Research Center for Obstetrics and Gynecology, Guangdong‐Hong Kong‐Macao Greater Bay Area Higher Education Joint Laboratory of Maternal‐Fetal Medicine The Third Affiliated Hospital, Guangzhou Medical University Guangzhou China; ^2^ Guangdong Medical Laboratory Animal Center Guangdong Provincial People's Hospital (Guangdong Academy of Medical Sciences), Southern Medical University Guangzhou China; ^3^ School of Basic Medical Sciences Southern Medical University Guangzhou China; ^4^ Institute of Comparative Medicine and Laboratory Animal Center Southern Medical University Guangzhou China; ^5^ Guangdong Provincial Key Laboratory of Large Animal Models for Biomedicine, School of Pharmacy and Food Engineering Wuyi University Jiangmen China

**Keywords:** central precocious puberty, gonadotropin inhibiting hormone (GnIH), gonadotropin releasing hormone (GnRH), *MKRN3*, rabbit

## Abstract

**Background:**

Makorin ring finger protein 3 gene (*MKRN3*) gene mutation is the most common genetic cause of central precocious puberty (CPP) in children. Due to the lack of ideal *MKRN3*‐modified animal model (*MKRN3*‐modified mice enter puberty only 4–5 days earlier than normal mice), the related research is limited.

**Methods:**

Therefore, the *MKRN3*‐modified rabbit was developed using CRISPR (clustered regularly interspaced short palindromic repeats) gene editing technology. The genotype identification and phenotype evaluation of *MKRN3*‐modified rabbits were carried out.

**Results:**

The first estrus of *MKRN3*‐modified female rabbits was observed ~27 days earlier than that of wild‐type female rabbits, with a typical CPP phenotype. This study found increased gonadotropin releasing hormone (GnRH) and decreased gonadotropin inhibiting hormone (GnIH) in the hypothalamus of the CPP rabbit model with MKRN3 gene mutation. Although this study failed to fully clarify the pathogenesis of CPP caused by *MKRN3* mutation, it found some differentially expressed genes and potential pathways through transcriptome sequencing.

**Conclusions:**

This study established a novel CPP model: paternal *MKRN3* gene‐modified rabbit. It is hoped that the establishment of this model will help researchers better understand, treat, and prevent CPP in the future.

## Graphical abstract

This study found increased gonadotropin releasing hormone (GnRH) and decreased gonadotropin inhibiting hormone (GnIH) in the hypothalamus of the central precocious puberty (CPP) rabbit model with MKRN3 gene mutation. It is suggested that the CPP caused by mkrtn3 mutation may be related to the activation of the GnRH pathway and the inhibition of the GnRH pathway, and the hypothalamic–pituitary–gonadal axis is activated under the joint action of the two pathways. FSH, follicle stimulating hormone; LH, luteinizing hormone.

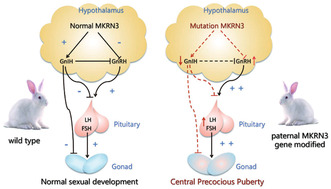

## 1 INTRODUCTION

Central precocious puberty (CPP) is a disease that causes precocious puberty in children due to premature activation of the hypothalamic–pituitary–gonadal axis (HPG axis).^1^ The incidence of CPP is 1/5000–1/10 000, 5–10 times more in girls than boys.^2^ CPP seriously endangers the physical and mental health of children, leading to short stature, obesity, low self‐esteem, and social barriers in adulthood, as well as increased risk of cardiovascular disease, metabolic disease, and cancer.^3^ CPP has a certain genetic predisposition. In 2013, the *New England Journal of Medicine* first reported that makorin ring finger protein 3 (*MKRN3*) gene can lead to CPP.^4^ In CPP with familial inheritance, the incidence of *MKRN3* mutation is the highest, about 30%.^5^ The human *MKRN3* gene is located on chromosome 15q11.2‐13 and has only one exon. It is a maternal imprinting gene located at the Prader Willi syndrome locus and can be expressed only in the paternal allele. Therefore, the patient inherits the *MKRN3* mutation of the father, which can cause CPP. At present, it has been found that a variety of *MKRN3* site mutations can cause CPP.^6^ The level of mkrn3 protein in the peripheral blood of CPP patients is significantly lower than that of normal people.^7^

Most researchers believe that *MKRN3* may have multiple inhibitory effects on HPG axis.^8^ Gonadotropin releasing hormone (GnRH) plays a core role in the upstream of the HPG axis. Most reports focus on the regulatory effect of *MKRN3* on *GNRH1* gene expression GnRH. However, Yellapragada et al. induced human‐induced pluripotent stem cells with *MKRN3* gene knockout to differentiate into *GNRH1* expressing neurons and found that it did not affect the expression level of GnRH.^9^ Ana Paula Abreu et al. proved that mkrn3 protein can also be expressed in normal mouse hypothalamic arcuate nucleus *KISS1* expressing neurons and in vitro proved that mkrn3 protein can inhibit *GNRH1* expressing neurons to secrete GnRH by inhibiting the promoter activity of human *KISS1* gene and *TAC3* gene.^10^ Other studies have found that mkrn3 protein may indirectly regulate *KISS1* gene and *GNRH1* gene through neuronalpentraxin 1 (*NPTX1*) in normal healthy mice.^11^ However, clinical studies have shown that there is no correlation between mkrn3 protein and nptx1 in human peripheral blood, suggesting that whether human mkrn3 protein regulates puberty by degrading *NPTX1* remains to be verified.^7^ Heras et al. found that miR‐30 in the hypothalamus of healthy rats can regulate puberty initiation by inhibiting *MKRN3* gene expression.^12^ Li et al. found that mkrn3 can ubiquitinate MBD3 protein, weaken the binding ability of MBD3 to the *GNRH1* promoter region and the recruitment ability to TET2, increase the methylation degree of *GNRH1*, and inhibit the expression of *GNRH1*.^13^ Recent studies have shown that *MKRN3* inhibits puberty onset via interaction with *IGF2BP1* and regulation of hypothalamic plasticity.^14^ The pathogenesis of CPP caused by MKRN3 has not been fully determined.

At present, in addition to the samples and data from clinical patients, the mechanism of CPP caused by *MKRN3* mutation mainly depends on the cell model. The first *MKRN3* gene–modified mouse CPP model was reported in 2020.^13^ However, *MKRN3* gene–modified mice enter puberty only 4–5 days earlier than normal mice,^13^ which is not conducive to research on the process of precocious puberty. Other makorin gene–modified animals, such as *MKRN1* gene–modified *Drosophila* ^15^ and *lep‐2* gene–modified *Caenorhabditis elegans*,^16^ have also been used to replace *MKRN3* to study CPP. This study successfully utilized CRISPR (clustered regularly interspaced short palindromic repeats) gene editing technology to cultivate a novel *MKRN3* gene–modified rabbit CPP model, which helps to overcome many animal model limitations in CPP research. Compared with mice, the development process of rabbits is slower, which is more suitable for an in‐depth observation of the occurrence and development of CPP and for treatment research.

## 2 METHODS

### 2.1 Animals

New Zealand rabbits used in this study were sourced from the Guangdong Medical Laboratory Animal Center in China. All experimental protocols were approved by the Ethics Committee for Animal Experiments of the Guangdong Medical Laboratory Animal Center (ethical review number: B202210‐6) and the Institutional Animal Care and Use Committee (IACUC) at Songshan Lake Pearl Laboratory Animal Science and Technology (IACUC approval number: S‐20200220‐01). This study follows procedures in accordance with ethical standards as formulated in the Helsinki Declaration of 1975 (revised 1983). All surgeries were performed under anesthesia, and every effort was made to minimize animal suffering. At present, the *MKRN3* gene–modified rabbits are being preserved by the Guangdong Medical Laboratory Animal Center, China (as noted in the article). If necessary, the institution can be contacted to obtain it.

### 2.2 Construction of the sgRNA and Cas9 mRNA

The single guide RNAs (sgRNAs) targeting the rabbit *MKRN3* genes were designed online (http://crispor.tefor.net/). The rabbit *MKRN3* gene has only one exon, and the sgRNA is designed to disrupt its C3HC4 zinc finger domain (Table S1). The synthesized oligonucleotide fragments RB‐MKRN3‐crRNA1 and sgRNAT7common (Table S2) were used to obtain the sgRNA in vitro transcription DNA templates through polymerase chain reaction (PCR) amplification. The PCR conditions consisted of a pre‐denaturation step at 98°C for 30 s, followed by 35 amplification cycles at 98°C for 10 s, 60°C for 30 s, and 72°C for 15 s, with a final extension step at 72°C for 10 min. After purification of the DNA template, the HiScribe T7 Quick High Yield RNA Synthesis Kit (E2050S, New England Biolabs, USA) was used to carry out the in vitro transcription of sgRNA according to the instructions. pST1374‐NLS‐flag‐linker‐Cas9 plasmids (44758, Addgene, USA) were digested with AgeI restriction endonuclease, followed by DNA purification and in vitro transcription, to synthesize CRISPR‐associated protein 9 (Cas9) messenger RNA (mRNA). The mMESSAGE mMACHINE T7 ULTRA Kit (AM1345, Life Technologies, USA) was used to synthesize Cas9 mRNA. The sgRNA and Cas9 mRNA were extracted using phenol/chloroform/isoamyl alcohol (25:24:1, pH <5.0) and then precipitated and purified using ethanol sodium acetate. Finally, the sgRNA and Cas9 mRNA were resuspended in a suitable amount of nuclease‐free water. The RNA concentration needs to be >1000 ng/μL, and it should be aliquoted into 5 μL/tube and stored at −80°C.

### 2.3 Synchronization of estrus and superovulation

First, the donor female rabbits in the non estrus phase were injected subcutaneously with 150 IU of pregnant mare serum gonadotropin (Hangzhou Animal Medicine Factory, China). Approximately 72 h later, they were mated with male rabbits and intravenously injected with 150 IU of human chorionic gonadotropin (Hangzhou Animal Medicine Factory, China). At the same time, female rabbits in the estrus phase were selected as surrogate mothers and intramuscularly injected with 15 IU of GnRH (Gonarelin, Hangzhou Animal Medicine Factory). After 20 h of mating, the donor female rabbits were anesthetized with sevoflurane and euthanized with carbon dioxide. The oviducts and uterus were then separated and flushed and examined for ova at 38.5°C with Dulbecco's phosphate‐buffered saline in vitro.

### 2.4 Microinjection and embryo transfer

A mixture of sgRNA (final concentration of 50 ng/μL) and Cas9 mRNA (final concentration of 100 ng/μL) was microinjected into the cytoplasm of rabbit zygotes. When the zygotes developed into the two‐cell stage, only one cell of the zygotes was injected. After microinjection, the embryos were placed in culture at 38.5°C for 30 min and then transferred to surrogate rabbits. Approximately 20–30 injected embryos were transferred to the recipient rabbits.

### 2.5 Genotype identification and off‐target analysis

Genotypes were identified as previously described.^17^ Briefly, the gene mutation of *MKRN3* was detected using PCR and Sanger sequencing. A drop of blood was collected from the rabbit, DNA was obtained by lysis, and the target gene loci were amplified using PCR with primers (Table S3). The PCR program was as follows: 95°C pre‐denaturation for 5 min; 94°C for 30 s, 62°C for 30 s, 72°C for 40 s, repeated 35 amplification cycles; and a final extension at 72°C for 5 min. Then, the PCR products were cloned using TA‐cloning vector and identified using Sanger sequencing. Potential CRISPR/Cas9 off‐target sites were predicted using the online tool (http://crispor.tefor.net/).

### 2.6 Breeding of *MKRN3* gene–modified rabbits

*MKRN3* is a maternally imprinted gene, and mutation of *MKRN3* from the paternal parent will cause CPP, so *MKRN3* gene–modified rabbits should be bred in accordance with the law of paternal inheritance. F_0_‐generation *MKRN3* chimeric male rabbits were crossed with wild‐type (WT) female rabbits (*MKRN3* ^m+/p+^) to obtain some F_1_‐generation *MKRN3* gene–modified rabbits (*MKRN3* ^m+/p–^). The F_1_‐generation male rabbits (*MKRN3* ^m+/p–^) with appropriate *MKRN3* mutation were selected for further breeding with WT female rabbits (*MKRN3* ^m+/p+^) to obtain the F_2_‐generation *MKRN3* gene–modified rabbits (*MKRN3* ^m+/p–^) and WT rabbits (*MKRN3* ^m+/p+^) (their ratio was ~1:1).

### 2.7 RT‐PCR and qPCR

Total RNA was isolated from hypothalamic brain tissue samples using Trizol reagent (15596026CN, Invitrogen, USA). Complementary DNA (cDNA) was synthesized using the PrimeScript RT reagent kit with genomic DNA (gDNA) Eraser (RR047A, Takara, Japan). The RT‐PCR primers of *MKRN3* gene are presented in Table S4. TB Green Premix Ex Taq II (RR820A, Takara) was used for qPCR. The qPCR primers are presented in Table S5.

### 2.8 Western blot

Protein was extracted from rabbit tissue using protein lysis buffer (PC101, Epizyme, China), and protein was quantified using the bicinchoninic acid assay method (P0012, Beyotime, China), followed by protein electrophoresis. After protein translocation, the antibody was incubated. The primary antibody used was antin mkrn3 antinbody (bs‐7149R, Bioss, China), and anti beta catin antibody was used as an internal reference (BM0627, Boster, China). The secondary antibody used was goat anti‐rabbit lgG (H + L) HRP (BS13278, Bioworld, USA) and goat anti‐mouse lgG (H + L) HRP (BS12478, Bioworld, USA).

### 2.9 Hormone‐level testing

About 2 mL of blood was collected from the artery of rabbit ear and placed in a coagulation vessel for 30 min. It was centrifuged at 4°C and 3000 rpm for 10 min, and the upper serum was collected for enzyme‐linked immunosorbent assay (ELISA) detection of follicle‐stimulating hormone (FSH) and luteinizing hormone (LH). The reagent kits used were the rabbit FSH ELISA detection kit (ml001657, Shanghai Enzyme‐linked Biotechnology Co., Ltd., China) and the rabbit LH ELISA Detection Kit (ml001779, Shanghai Enzyme‐linked Biotechnology Co., Ltd).

### 2.10 Hematoxylin and eosin staining, immunohistochemistry, and immunofluorescence

The rabbit organs and tissues were first fixed in 4% paraformaldehyde and then cut into paraffin sections. The sections were stained with hematoxylin and eosin (H&E) for histological analysis and analyzed using immunohistochemistry (IHC) and immunofluorescence (IF). In IHC and IF, anti‐ZNF127 antibody (bs‐7149R, Bioss) was used to identify mkrn3, anti‐GNRH/LHRH antibody (bs‐10369R, Bioss) was used to identify GnRH, anti‐NPVF/RFRP antibody (bs‐19339R, Bioss) was used to identify gonadotropin inhibiting hormone (GnIH), and anti‐Npffr1 antibody (bs‐12018R, Bioss) was used to identify Npffr1.

### 2.11 Bimolecular fluorescence complementation

First, different rabbit *MKRN3* DNA segments were obtained, including intact *MKRN3*, *MKRN3* with a missing first C3H domain (CCCH1), *MKRN3* with a missing second C3H domain (CCCH2), *MKRN3* with a missing third C3H domain (CCCH3), *MKRN3* with a missing CH domain, and *MKRN3* with a missing C3HC4 domain. Then, different rabbit *MKRN3* DNA fragments were fused with GFP‐N173 and GFP‐C155 fragments, respectively, and inserted into the pCDNA3.1 (+) expression vector to construct different bimolecular fluorescence complementation (BiFC) plasmids pairs (Table S6) to detect the binding of mkrn3 homodimers. Instantly different BiFC plasmid pairs were transfected into HEK‐293T cells at 1:1 concentration, and green excitation fluorescence was observed 48 h after transfection. The more fluorescence there is, the stronger the binding ability of the homodimer.

### 2.12 Transcriptome RNA sequencing and analysis

Different groups of rabbit hypothalamic tissues were collected and stored in liquid nitrogen; then total RNA was extracted. Transcriptome RNA sequencing (RNA‐seq) experiments and data analyses were performed by DIATRE Biotechnology (Shanghai, China). Briefly, the sequencing platform used was Illumina Nova Seq, the sequencing mode was PE150, and the reference species data used was OryCun2.0 (Ensembl database). Cufflinks software was used to compute the gene expression level and difference.^18^ DEseq2 was further used to analyze the data.^19^ The raw sequence data reported in this paper have been deposited in the Genome Sequence Archive (GSA)^20^ in the National Genomics Data Center,^21^ China National Center for Bioinformation/Beijing Institute of Genomics, Chinese Academy of Sciences (GSA: CRA018509) that are publicly accessible at https://ngdc.cncb.ac.cn/gsa.

### 2.13 Quantification and statistical analysis

Student's *t*‐test was used for statistical analysis between every two groups. Data are expressed as mean ± standard error of the mean. Statistical analyses were performed using GraphPad Prism software. The *p‐*value <0.05 was considered as statistically significant.

## 3 RESULTS

### 3.1 Production of rabbits with paternal *MKRN3* ‐modified genes

**FIGURE 1:**
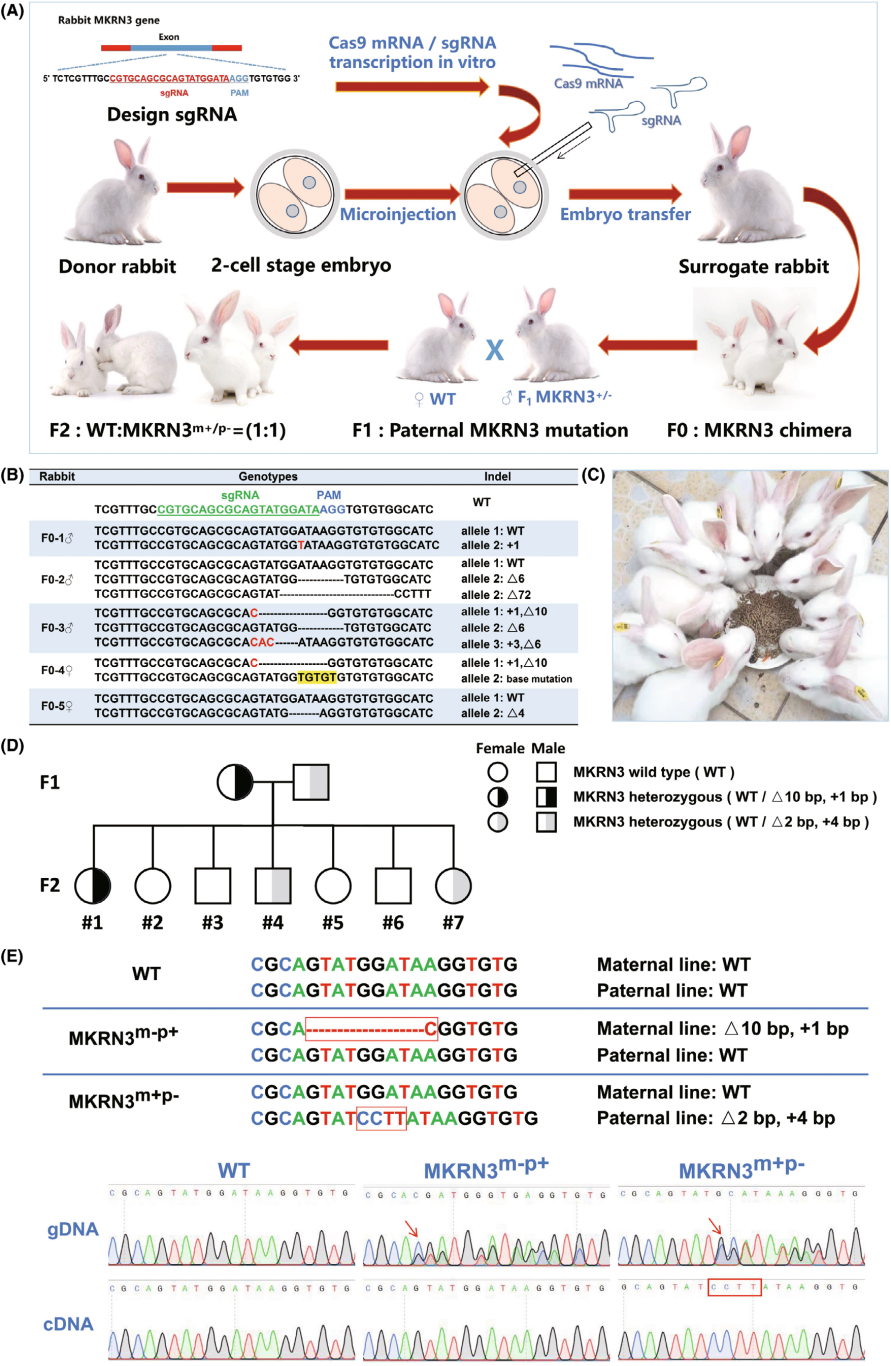
Breeding of paternal *MKRN3* gene–modified rabbits. (A) Technology strategy: Production of *MKRN3* chimeric rabbit by CRISPR/Cas9 cytoplasmic microinjection of two‐cell‐stage embryos. (B) Preliminary genotype identification of F_0_ generation. (C) Images of F_2_‐generation rabbits. (D) Breeding of the heterozygous F_1_ generation carrying the mutant *MKRN3* gene. (E) cDNA, complementary DNA; gDNA, genomic DNA. DNA Sanger sequencing results: Even though the individual's gDNA shows that it has different *MKRN3* double alleles (shown by a red arrow), *MKRN3* can still maintain the characteristics of maternal imprinted genes and is expressed only in paternal alleles (shown by a red box).

This study conducted a comparison of the mkrn3 protein sequences between humans and various common laboratory animals, including mouse, rat, rabbit, pig, sheep, dog, cat, and rhesus macaque (Figure S1). It can be seen that apart from rhesus macaque, the mkrn3 protein in rabbits has a high similarity to human mkrn3 (84.20% similarity). Referring to the human mkrn3 protein structure and mutation sites (Figure S2A), we analyzed the *MKRN3* gene in rabbits (Table S1). Similar to the human *MKRN3* gene, rabbit *MKRN3* also has only one exon. The C3HC4 domain of the mkrn3 protein is related to the activity of E3 ubiquitin ligase and may be related to the stability of some puberty initiation–related proteins, so this study targeted this domain. sgRNAs were designed in front of the corresponding gene loci to cause frameshift mutations in the *MKRN3* gene (Figure S2B). Clinically, the CPP caused by human *MKRN3* mutation is derived from paternal mutation, and double allele mutation is very rare. Therefore, this study adopted a different strategy (Figure 1A): injected only one two‐cell‐stage embryos, obtained F_0_‐generation *MKRN3* gene–modified chimera (Figure 1B), and further bred more offspring (Figure 1C). To facilitate offspring genotype identification, this study selected the *MKRN3* gene–modified rabbits with a deletion of 72 bp for conservation and breeding (the mkrn3 protein lacks only the C3HC4 domain) for CCP research (Figure S3).

To observe the gene inheritance characteristics of *MKRN3*, it bred the heterozygous F_1_ generation carrying the mutant *MKRN3* gene. The female rabbit (*MKRN3* genotype: WT; △10 bp, +1 bp) mated with the male rabbit (*MKRN3* genotype: WT; △2 bp, +4 bp) (Figure 1D). Among the F_2_ generations obtained, four rabbits were WT, one rabbit carried maternal *MKRN3* mutation (*MKRN3* ^m‐p+^, maternal: △10 bp, +1 bp; paternal: WT), and the remaining two rabbits carried the paternal *MKRN3* mutation (*MKRN3* ^m+p–^, maternal: WT; paternal: △2 bp, +4 bp) (Figure 1D). To verify whether the modified *MKRN3* maintains the inheritance pattern of maternal imprinting genes, gDNA and cDNA were extracted from F_2_‐generation rabbits, and then DNA Sanger sequencing was performed after PCR amplification of the *MKRN3* locus (Figure 1E). The cDNA sequencing of the rabbit carrying maternal *MKRN3* mutation (*MKRN3* ^m–p+^) showed WT, indicating that maternal *MKRN3* is not expressed in vivo. The cDNA sequencing of the rabbit carrying the paternal *MKRN3* mutation (*MKRN3* ^m+p–^) showed Δ2 bp, +4 bp mutations, consistent with the paternal mutation type, indicating in vivo expression of paternal *MKRN3* (Figure 1E). This indicates that the modified *MKRN3* can still maintain the characteristics of maternal imprinting genes and is expressed only in paternal alleles.

### 3.2 Paternal *MKRN3* –modified rabbits exhibit CPP phenotype

**FIGURE 2:**
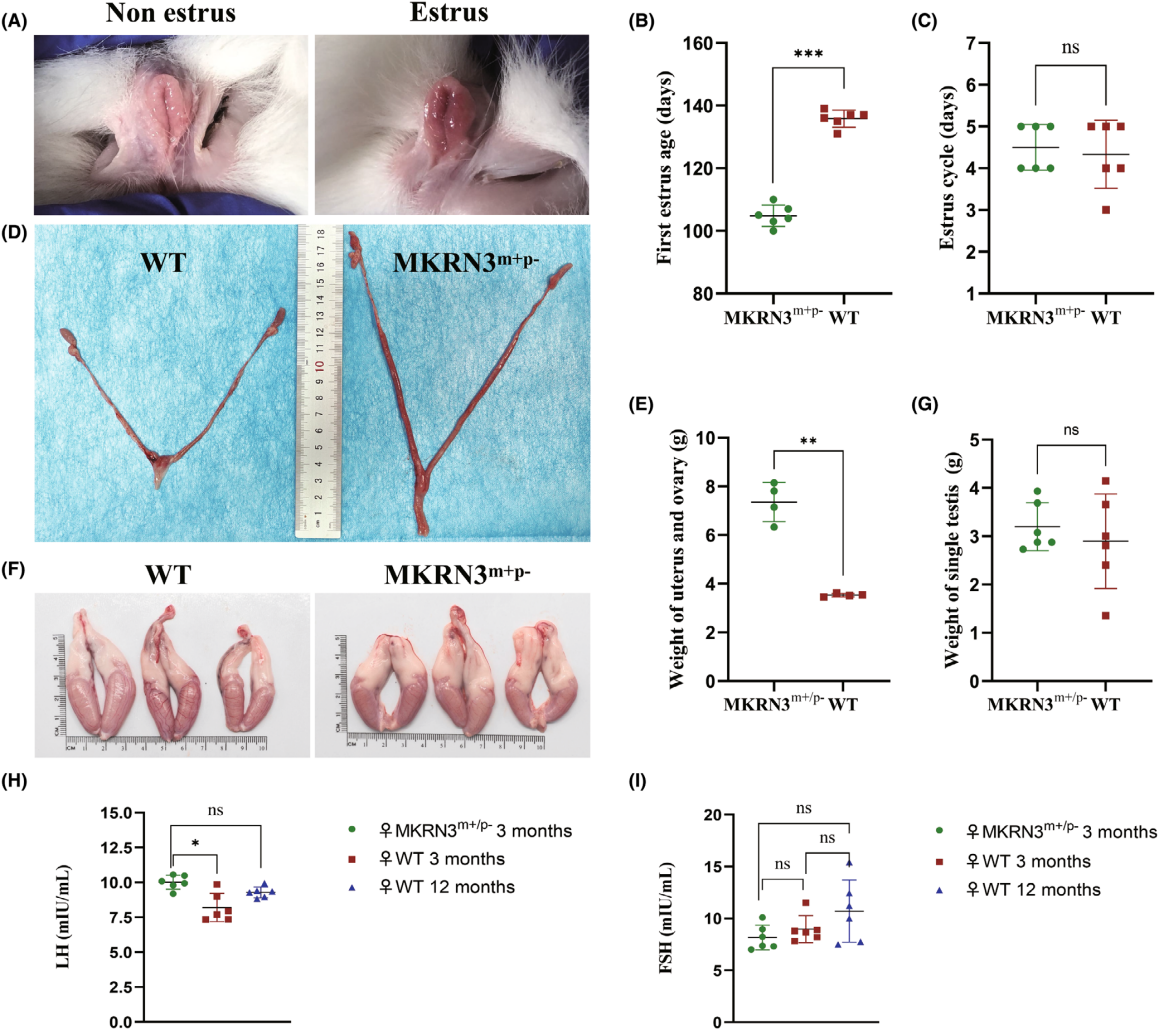
Paternal *MKRN3*‐modified rabbits exhibit CPP (central precocious puberty) phenotype. (A) The color of the vulva can be used to determine whether the vulva is in estrus. When the vulva is red or purple red, it is in the estrus phase, but it is light pink in the non‐estrus phase. The first appearance of purple in the vagina of *MKRN3* gene–modified rabbits was younger than that of wild type (WT). (B) Comparison of the first estrus age of *MKRN3* gene–modified female rabbits with the same age WT (*n* = 6). ns: *p* > 0.05; *p ≤ 0.05; **p ≤ 0.01; ***p ≤ 0.001. (C) Comparison of the estrus cycle of *MKRN3* gene–modified female rabbits with the same age WT (*n* = 6). ns: *p*> 0.05; *p ≤ 0.05; **p ≤ 0.01; ***p ≤ 0.001. (D) The overall appearance of uterus, fallopian tube, and ovary in 3‐month‐old rabbits. (E) Statistical analysis of weight of uterus and ovary (*n* = 4). ns: *p*> 0.05; *p ≤ 0.05; **p ≤ 0.01; ***p ≤ 0.001. (F) The overall appearance of testis in 3‐month‐old rabbits. (G) Statistical analysis of single testicular weight (*n* = 6). ns: *p* > 0.05; *p ≤ 0.05; **p ≤ 0.01; ***p ≤ 0.001. (H, I) The serum LH (luteinizing hormone) or FSH (follicle‐stimulating hormone) levels of 3‐month‐old *MKRN3* gene–modified rabbits were compared with those of the same‐age female rabbits and 12‐month‐old female rabbits (*n* = 6). ns: *p* > 0.05; *p ≤ 0.05; **p ≤ 0.01; ***p ≤ 0.001. *MKRN3* ^m+/p–^, paternal mutant *MKRN3*‐modified rabbits; WT, wild‐type rabbits.

By observing the color changes in the external genitalia (Figure 2A) and vaginal cell smears daily to determine estrus,^17^ the age and cycle of estrus in female rabbits were recorded. The first estrus age of *MKRN3*‐modified female rabbits was 107.8 ± 3.9 days, which was significantly earlier than that of WT female rabbits (134.0 ± 4.6 days) (*p* < 0.01) (Figure 2B). The estrus cycle of *MKRN3*‐modified female rabbits is normal (Figure 2C). In fact, at age 90 days, *MKRN3*‐modified female rabbits exhibited significantly earlier uterine development and a much larger uterus compared to WT mice of the same age (Figures 2D and 4E). However, at age 90 days, there was no significant difference in testicular volume between *MKRN3*‐modified male rabbits and WT male rabbits of the same age (Figure 2F,G). Compared with WT female rabbits of the same age, the serum LH level of 3‐month‐old *MKRN3*‐modified female rabbits was higher (Figure 2H), similar to that of 12‐month‐old WT female rabbits, whereas the serum FSH level was normal (Figure 2I). There was no significant difference in body weight between 3‐month‐old *MKRN3* gene–modified rabbits and WT rabbits of the same age and sex (Figure S4).

**FIGURE 3:**
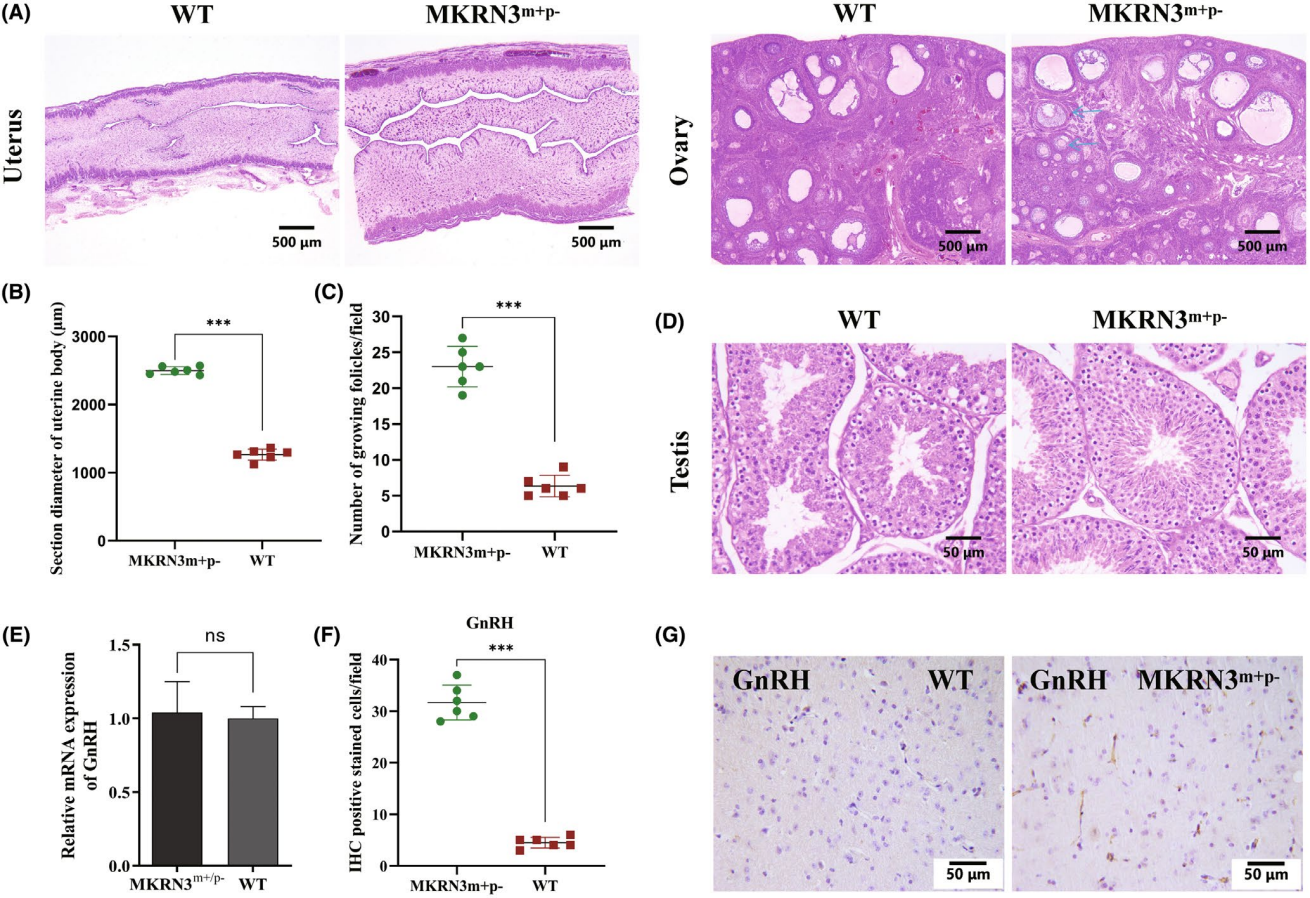
CPP (central precocious puberty) pathological examination of paternal *MKRN3*‐modified rabbits. (A) H&E (hematoxylin and eosin) staining results: It can be seen that the uterine wall thickness of 3‐month‐old *MKRN3*‐modified rabbits is about twice that of WT rabbits of the same age; the number of developing follicles (blue arrow) in the ovaries of 3‐month‐old *MKRN3*‐modified rabbits is significantly higher than that of WT rabbits of the same age. Scale bar = 500 μm. (B) Statistical analysis of uterine body section diameter (corresponding to Figure 3A) (*n* = 6). ns: *p* > 0.05; *p ≤ 0.05; **p ≤ 0.01; ***p ≤ 0.001. (C) Statistics of the number of follicles in ovarian development (corresponding to Figure 3A) (*n* = 6). ns: *p* > 0.05; *p ≤ 0.05; **p ≤ 0.01; ***p ≤ 0.001. (D) H&E staining results: Compared with the WT of the same age, it can be seen that the testes of 3‐month‐old *MKRN3* gene–modified rabbits have more mature sperm development. Scale bar = 50 μm. (E) The qPCR results of the hypothalamic GnRH (gonadotropin releasing hormone) mRNA levels between 3‐month‐old *MKRN3* gene–modified rabbits and WT rabbits of the same age (*n* = 6). ns: *p* > 0.05; *p ≤ 0.05; **p ≤ 0.01; ***p ≤ 0.001. (F) IHC (immunohistochemistry) staining results of GnRH in the hypothalamus. Scale bar = 50 μm. (G) Statistics of GnRH‐positive cells in IHC staining results (corresponding to Figure 3F) (*n* = 6). ns: *p* > 0.05; *p ≤ 0.05; **p ≤ 0.01; ***p ≤ 0.001. *MKRN3* ^m+/p–^, paternal mutant *MKRN3*‐modified rabbits; WT, wild‐type rabbits.

Pathology showed that the uterine wall of 3‐month‐old *MKRN3*‐modified female rabbits was thicker and more mature than that of WT female rabbits of the same age (Figure 3A,B). More growing follicles were observed in the ovaries of 3‐month‐old *MKRN3*‐modified female rabbits (Figure 3A,C). Pathology showed that compared with WT male rabbits of the same age, *MKRN3*‐modified male rabbits had more mature sperm in the testis (Figure 3D). Compared with WT rabbits of the same age, 3‐month‐old *MKRN3*‐modified rabbits exhibited no difference in GnRH mRNA levels in the hypothalamus (*p* > 0.05) (Figure 3E). However, IHC showed that the expression level of GnRH in 3‐month‐old *MKRN3*‐modified rabbits significantly increased (Figure 3F,G).

### 3.3 *MKRN3* ‐modified rabbit brain tissue shows increased expression of GnRH, and decreased expression of GnIH and neuropeptide FF receptor 1

**FIGURE 4:**
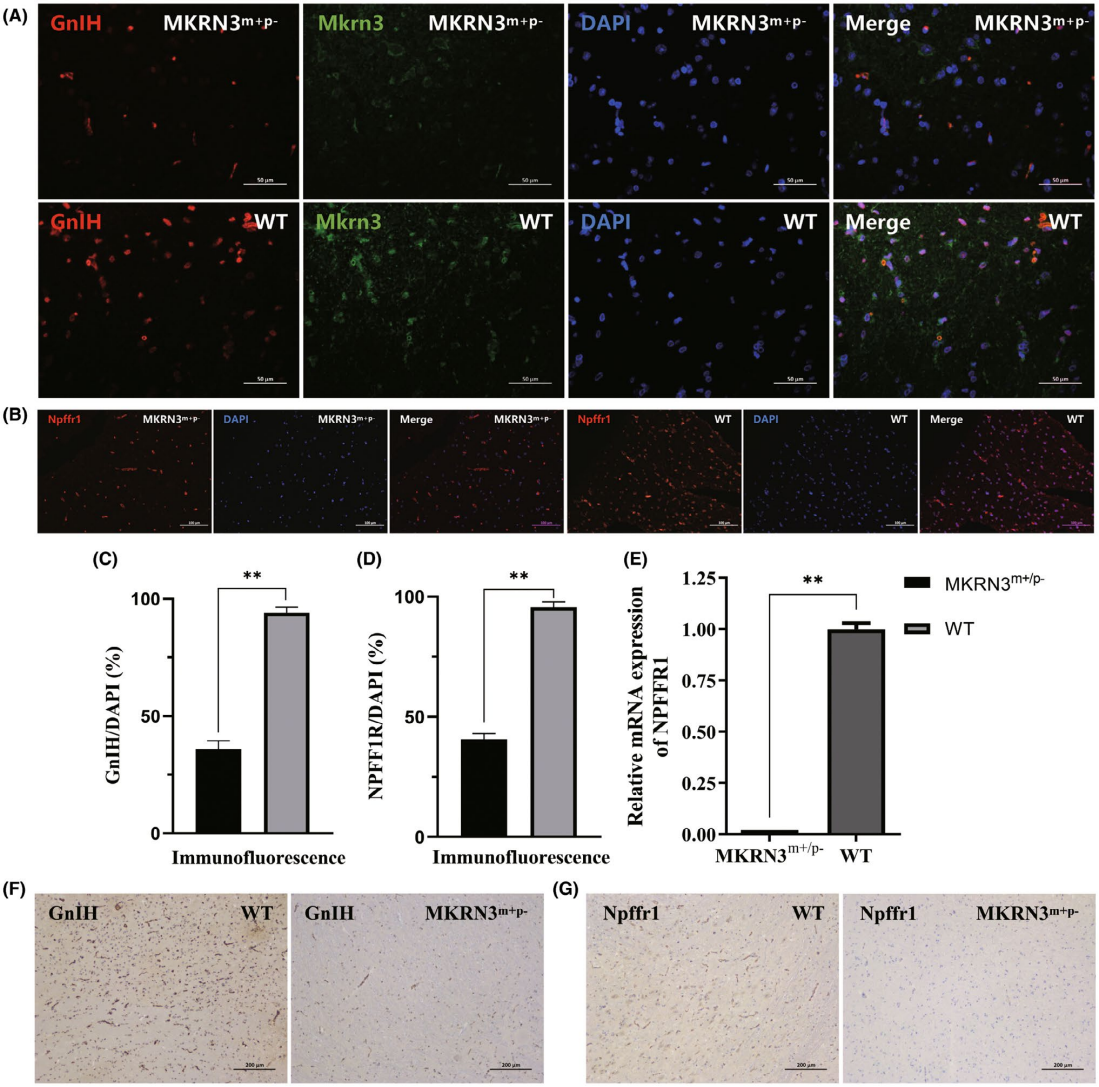
Comparison of GnRH (gonadotropin releasing hormone) and Npffr1 expression levels between MKRN3‐modified rabbits and wild‐type (WT) rabbits. (A) Hypothalamic IF (immunofluorescence) staining results. DAPI (2‐(4‐Amidinophenyl)‐6‐indolecarbamidine dihydrochloride): Nuclear staining. Scale bar = 50 μm. (B) Pituitary IF staining results. DAPI: Nuclear staining. Scale bar = 100 μm. (C) The IF result statistics of GnIH (gonadotropin inhibiting hormone) (*n* = 6) (corresponding to Figure 4A). ns: *p*> 0.05; *p ≤ 0.05; **p ≤ 0.01; ***p ≤ 0.001. (D) The IF results of Npffr1 were statistically analyzed (*n* = 6) (corresponding to Figure 4B). ns: *p* > 0.05; *p ≤ 0.05; **p ≤ 0.01; ***p ≤ 0.001. (E) qPCR results: Detection of mRNA levels of *NPFFR1* in the hypothalamus of *MKRN3*‐modified rabbits and WT rabbits (*n* = 6). ns: *p* > 0.05; *p ≤ 0.05; **p ≤ 0.01; ***p ≤ 0.001. (F) Hypothalamic IHC (immunohistochemistry) staining results. Scale bar = 200 μm. (G) Pituitary IHC staining results. Scale bar = 200 μm. *MKRN3* ^m+/p–^: 3‐month‐old paternal mutant *MKRN3*‐modified rabbits; WT, 3‐month‐old WT rabbits.

Based on the strong inhibitory effect of GnIH on the HPG axis, this study also tested GnIH and its receptor neuropeptide FF receptor 1 (Npffr1). IF results showed that the GnIH expression in the hypothalamus was also significantly reduced (Figure 4A), with an expression level only about 39.1% of that of WT rabbits of the same age (Figure 4C). The Npffr1 protein in the pituitary gland of 3‐month‐old *MKRN3*‐modified rabbits was significantly reduced (Figure 4B), and its expression level was only about 39.8% of that of WT rabbits of the same age (Figure 4D). Compared with WT rabbits of the same age, the expression level of *NPFFR1* mRNA in the brain tissue of 3‐month‐old *MKRN3* gene–modified rabbits was also significantly reduced (Figure 4E). Similarly, the IHC results also indicated that the expression levels of GnIH and Npffr1 in 3‐month‐old *MKRN3*‐modified rabbits were significantly reduced compared to WT rabbits of the same age (Figure 4F,G).

### 3.4 Transcriptome sequencing analysis between paternal *MKRN3* ‐modified rabbits and WT rabbits

**FIGURE 5:**
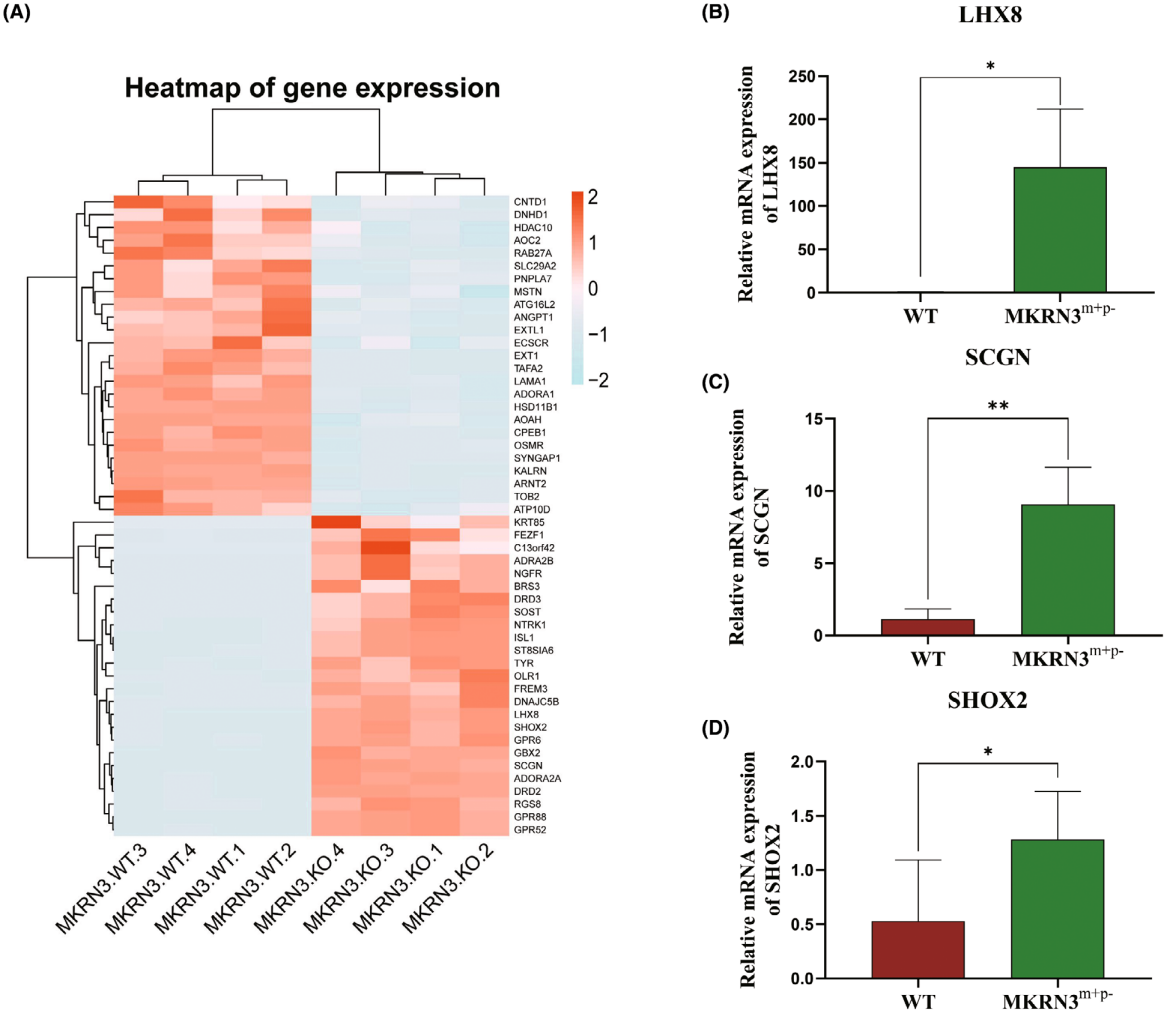
The heat map of DEGs (differentially expressed genes). (A) Transcriptome sequencing analysis between paternal *MKRN3*‐modified rabbit hypothalamics and WT rabbit hypothalamics. The heat map of DEGs is shown. (B–D) The RT‐qPCR results of several candidate genes in DEGs. ns: *p* > 0.05; *p ≤ 0.05; **p ≤ 0.01; ***p ≤ 0.001. The raw sequence data (GSA [Genome Sequence Archive]: CRA018509) are publicly accessible at https://ngdc.cncb.ac.cn/gsa.

**FIGURE 6:**
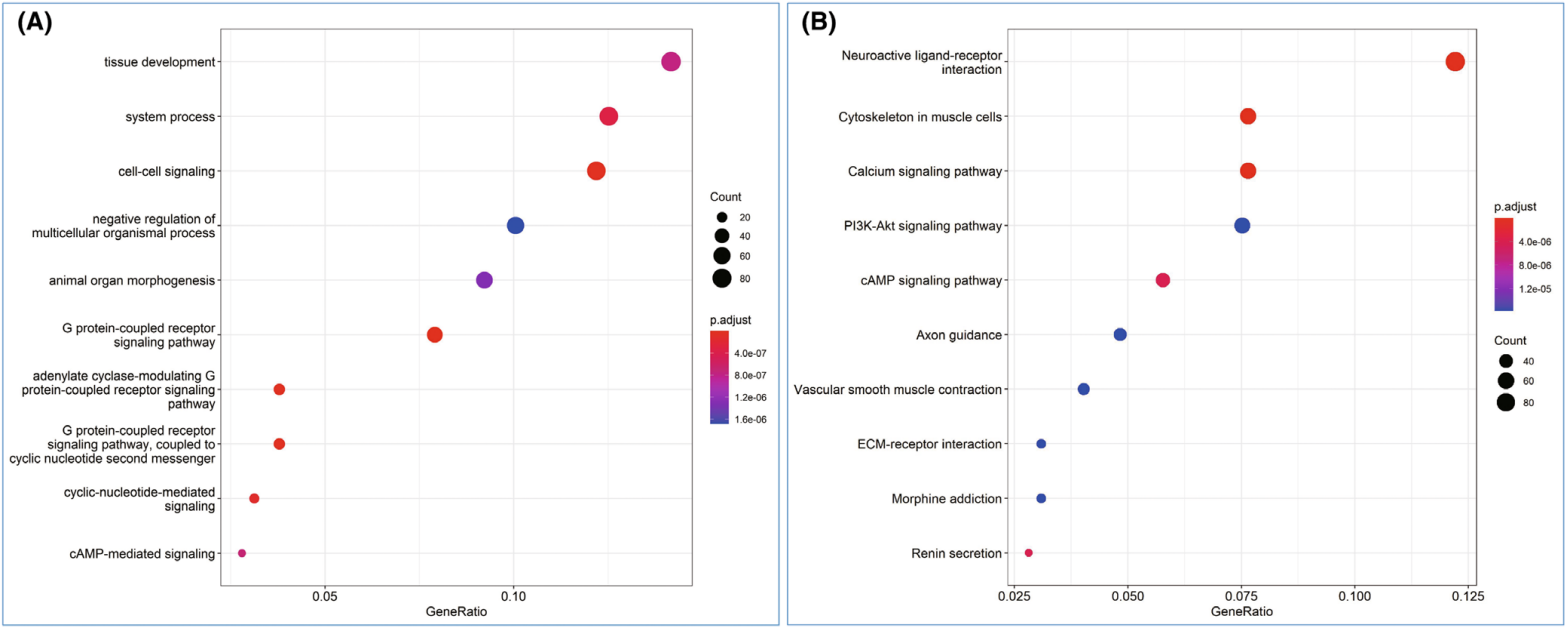
GO (Gene Ontology) enrichment analysis and KEGG (Kyoto Encyclopedia of Genes and Genomes) analysis. (A) GO enrichment analysis chart of significantly differentially expressed genes (DEG) between paternal *MKRN3*‐modified rabbit hypothalamics and wild‐type (WT) rabbit hypothalamics. (B) KEGG analysis chart of significantly DEGs between paternal *MKRN3*‐modified rabbit hypothalamics and WT rabbit hypothalamics. The raw sequence data (GSA [Genome Sequence Archive]: CRA018509) that are publicly accessible at https://ngdc.cncb.ac.cn/gsa.

Transcriptome sequencing analysis showed that there were many differentially expressed genes between 3‐month‐old *MKRN3* gene–modified rabbits (*MKRN3* ^m+/p–^) and WT rabbits of the same age. The heat map of differentially expressed genes (DEG) was used to present the distribution of DGEs (Figure 5A). Transcriptome sequencing analysis showed that *MKRN3* mutation leads to significant changes in the expression of many transcription factors. The RT‐qPCR results of several candidate genes in DEGs are highly consistent with the sequencing results (Figure 5B–D). Gene ontology enrichment analysis and Kyoto Encyclopedia of Genes and Genomes analysis were used to uncover the potential enriched pathways (Figure 6A,B). The dual fluorescence complementation experiment found that *MKRN3* protein can form dimers in vitro (Figure S5), and it was found that the homodimer binding ability of some *MKRN3* mutants increased instead (Figure S6).

## 4 DISCUSSION

This study successfully obtained the *MKRN3*‐modified rabbit CPP model, which reproduced the human genetic CPP phenotype. Compared with *MKRN3* gene–modified mice, *MKRN3* gene–modified rabbits have longer sexual maturity time and produce larger individuals, which is convenient for observing estrus and repeatedly detecting serum hormone levels, which is helpful to better understand the mechanism of puberty initiation and CPP occurrence and development, and to carry out drug treatment evaluation tests. This breaks through the limitation of CPP model research. It was observed that the initial estrus age of *MKRN3* gene–modified rabbits was about 1 month earlier than that of WT rabbits. The uterus of 3‐month‐old *MKRN3* gene–modified rabbits was much more mature than that of WT rabbits of the same age, suggesting that *MKRN3* rabbits actually may have had an unobservable onset of puberty at a younger age. Like the human CPP, female phenotypes in early puberty are more obvious and easier to observe, whereas male phenotypes are more secretive and harder to observe. Unlike humans, rabbits are stimulated ovulating animals and need mating stimulation to ovulate. This may explain why there is no significant difference in FSH levels between 3‐month‐old *MKRN3*‐modified rabbits and WT rabbits of the same age (Figure 2I). It is worth mentioning that among common experimental animals, besides monkeys, the mkrn3 protein sequence of rabbits is most similar to that of human mkrn3 protein (Figure S1). This makes rabbits more suitable as *MKRN3* mutant CPP models.

If the mechanism and role of the *MKRN3* gene in adolescent initiation can be elucidated, as well as how *MKRN3* gene mutations lead to CPP, it would be of great significance and provide guidance strategies for the treatment of CPP children. However, this mechanism is quite complex. In fact, the question of what triggers puberty has yet to be fully elucidated, and it has been listed as one of the most cutting‐edge scientific challenges by the journal *Science*.^22^ Despite our efforts to determine the mechanism, we can only uncover fragmented potential mechanisms. The main speculated mechanisms include the following two aspects. (1) General mechanism: the reason for CPP occurrence is the premature activation of the HPG axis. The HPG axis is regulated by many known or unknown nerves and hormones, among which GnRH is an important HPG axis–activating hormone, whereas GnIH is an important HPG axis–inhibiting hormone, and the two are mutually antagonistic. When *MKRN3* is inactivated, it causes an increase in GnRH expression and a decrease in GnIH expression, leading to an imbalance between these two hormones and premature activation of the HPG axis, resulting in CPP. Many reported studies have focused on the mechanism of how *MKRN3* mutations promote GnRH expression,^23^, ^24^ ignoring the important inhibitory role of GnIH in the HPG axis. Therefore, this study validated the changes in GnRH and GnIH after developing *MKRN3* gene–modified rabbits, emphasizing the importance of antagonizing these two pathways. Loss of *MKRN3* function resulted in a marked decrease in the expression of GnIH and its receptor Npffr1, which has not been reported before. (2) Molecular mechanism: the preliminary experiments of this study did not show that mkrn3 protein could directly bind to GnRH and GnIH, nor could it directly bind to upstream sequences of genes corresponding to GnRH and GnIH regulation. Based on the zinc finger protein structure and E3 ubiquitin ligase structure of mkrn3,^6^ we speculate that mkrn3 plays different roles in the cytoplasm and nucleus. Transcriptome sequencing analysis showed that *MKRN3* mutation leads to significant changes in the expression of many transcription factors (Figure 5A). The dual fluorescence complementation experiment found that mkrn3 protein can form dimers in vitro (Figure S5), and it was found that the homodimer binding ability of some *MKRN3* mutants increased instead (Figure S6). Therefore, it is speculated that mutations in the *MKRN3* gene may affect the ability of mkrn3 to form heterodimers with other transcription factors, thereby affecting its binding and regulatory abilities with various target DNA or RNA to varying degrees, leading to the differential hormone expression of GnRH and GnIH. This study detected that although the polypeptide‐level expression of GnRH in 3‐month‐old *MKRN3*‐modified rabbits increased (Figure 3F,G), the *GnRH* mRNA level was not significantly different (Figure 3E), suggesting that mkrn3 may be involved in other potential mechanisms such as stability regulation, posttranscriptional modification, or translation regulation of *GnRH* mRNA. There have been reports on the E3 ubiquitin ligase structure and function of mkrn3 protein. Mkrn3 can ubiquitinate MBD3 protein, weaken the binding ability of MBD3 to the *GNRH1* promoter region and the recruitment ability to TET2, increase the methylation degree of *GNRH1*, and inhibit the expression of *GNRH1*.^13^ In addition, mkrn3‐mediated ubiquitination of poly(A)‐binding proteins modulates the stability and translation of *GNRH1* mRNA in mammalian puberty.^25^

Other studies have reported that *MKRN3* inactivation can promote lung cancer progression,^26^ but in this study, no lung cancer was observed in rabbits with *MKRN3* gene mutations. Except for a few F_0_‐generation rabbits with abnormal development (Figure S7) (possibly due to complete knockout of the double allele at the imprinting gene locus, causing methylation abnormalities at that locus), the F_1_, F_2_, and F_3_ generations of rabbits with *MKRN3* gene mutations are all very healthy, and several of them are already 3 years old. The F_0_ chimeras obtained by two‐cell‐stage embryo editing in this study are healthy and conducive to the reproduction of offspring, which can provide some experience for the subsequent cultivation of gene editing animal models related to other imprinted genes.

In general, this study developed a novel rabbit model of CPP by modifying the imprinted gene *MKRN3*. Due to the complex mechanism of adolescent development, this study cannot fully determine the mechanism of *MKRN3* mutation leading to CPP. However, the model provides us with some new insights. We hope that these models can help to further understand the pathogenesis of CPP caused by *MKRN3* mutations and the development and preclinical evaluation of CPP drugs, and have good application prospects.

## AUTHOR CONTRIBUTIONS

**Bangzhu Chen:** Conceptualization; data curation; funding acquisition; investigation; project administration; writing – original draft. **Xing Ye:** Conceptualization; data curation; investigation; methodology. **Lihao Chen:** Data curation; investigation; methodology. **Tianping Liu:** Formal analysis; methodology; validation. **Guiling Li:** Investigation; software; supervision; validation. **Chula Sa:** Data curation; investigation; methodology; writing – original draft. **Juan Li:** Data curation; formal analysis; methodology; software; supervision. **Ke Liu:** Project administration; resources; supervision; validation. **Weiwang Gu:** Conceptualization; funding acquisition; project administration; resources; supervision; validation. **Gang Wang:** Conceptualization; funding acquisition; supervision; validation; writing – review and editing.

## FUNDING INFORMATION

This research was supported by the National Natural Science Foundation of China (grant no.: 82101937); the Guangdong Medical Science and Technology Research Fund Project, China (grant no.: B2024069); and the Guangzhou Science and Technology Plan Project, China (grant no.: SL2023A04J02229, assignment no.: 2024A04J4923).

## CONFLICT OF INTEREST STATEMENT

The authors declare no conflict of interest.

## ETHICS STATEMENT

This study were approved by the Ethics Committee for Animal Experiments of the Guangdong Medical Laboratory Animal Center (ethical review number: B202210‐6) and the Institutional Animal Care and Use Committee (IACUC) at Songshan Lake Pearl Laboratory Animal Science and Technology (IACUC approval number: S‐20200220‐01).

## Supporting information


Figure S1.

Figure S2.

Figure S3.

Figure S4.

Figure S5.

Figure S6.

Figure S7.

Table S1.

Table S2‐S5.

Table S6.

## Data Availability

The raw sequence data reported in this paper have been deposited in the Genome Sequence Archive in National Genomics Data Center, China National Center for Bioinformation / Beijing Institute of Genomics, Chinese Academy of Sciences (GSA: CRA018509) that are publicly accessible at https://ngdc.cncb.ac.cn/gsa.
