# Supplementary material for: A novel model of central precocious puberty disease: Paternal MKRN3 gene–modified rabbit
Source: Animal Model Exp Med. 2025 Jan 24;8(3):511–22. doi: 10.1002/ame2.12544 (PMC11904109; doi:10.1002/ame2.12544)
Supplement: Supplementary file 1 — Figure S1. [file AME2-8-511-s001.pdf]

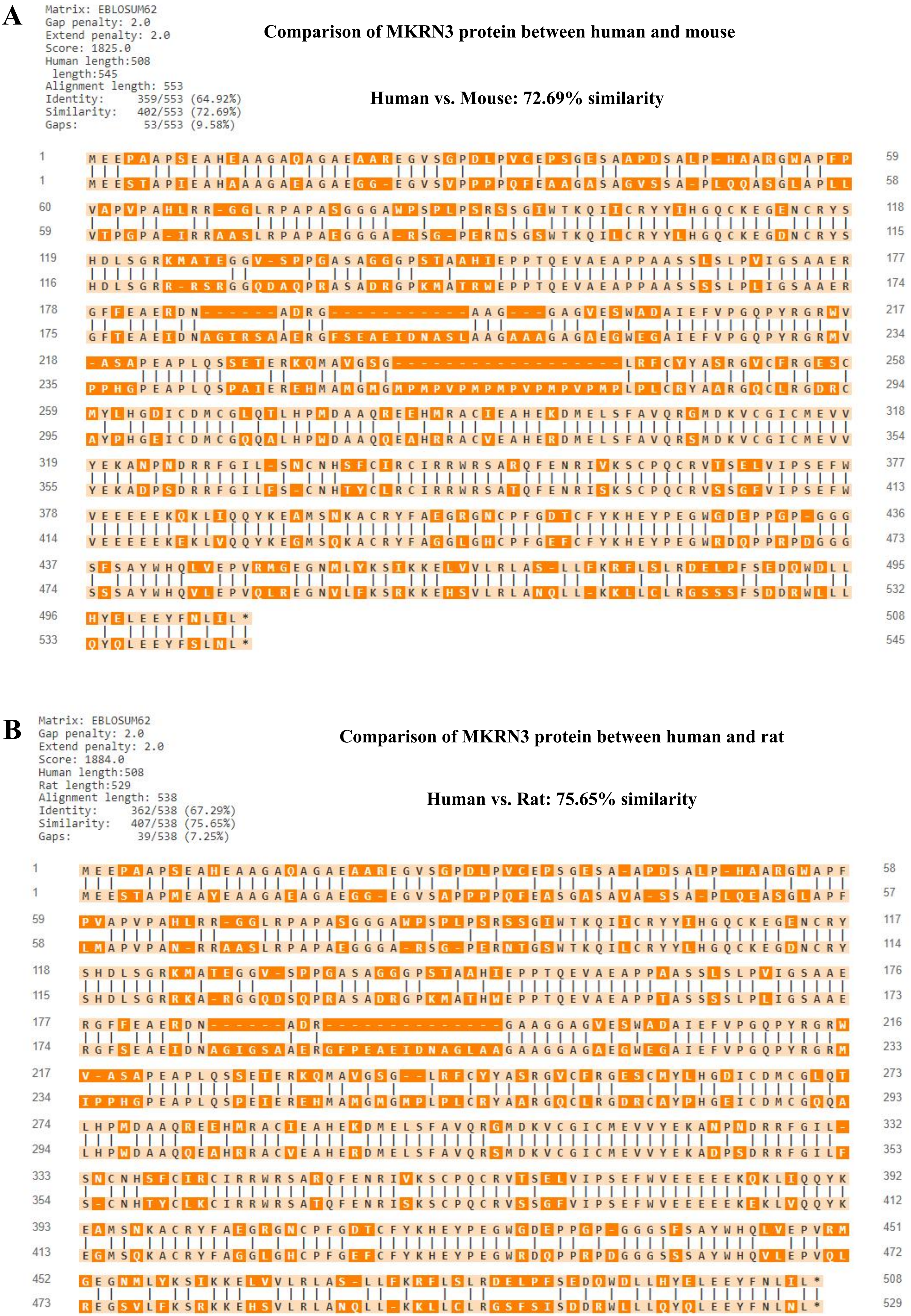
A
Matrix: EBLOSUM62
Gap penalty: 2.0
Extend penalty: 2.0
Score: 1825.0
Human length:508
length:545
Alignment length: 553
Identity: 359/553 (64.92%)
Similarity: 402/553 (72.69%)
Gaps: 53/553 (9.58%)
Comparison of MKRN3 protein between human and mouse
Human vs. Mouse: 72.69% similarity
B
Matrix: EBLOSUM62
Gap penalty: 2.0
Extend penalty: 2.0
Score: 1884.0
Human length:508
Rat length:529
Alignment length: 538
Identity: 362/538 (67.29%)
Similarity: 407/538 (75.65%)
Gaps: 39/538 (7.25%)
Comparison of MKRN3 protein between human and rat
Human vs. Rat: 75.65% similarity

C

```
Matrix: EBLOSUM62
Gap penalty: 2.0
Extend penalty: 2.0
Score: 2094.0
Human length:508
Rabbit length:516
Alignment length: 519
Identity:      390/519 (75.14%)
Similarity:    437/519 (84.20%)
Gaps:           14/519 (2.70%)
```

Comparison of MKRN3 protein between human and rabbit

Human vs. Rabbit: 84.20% similarity

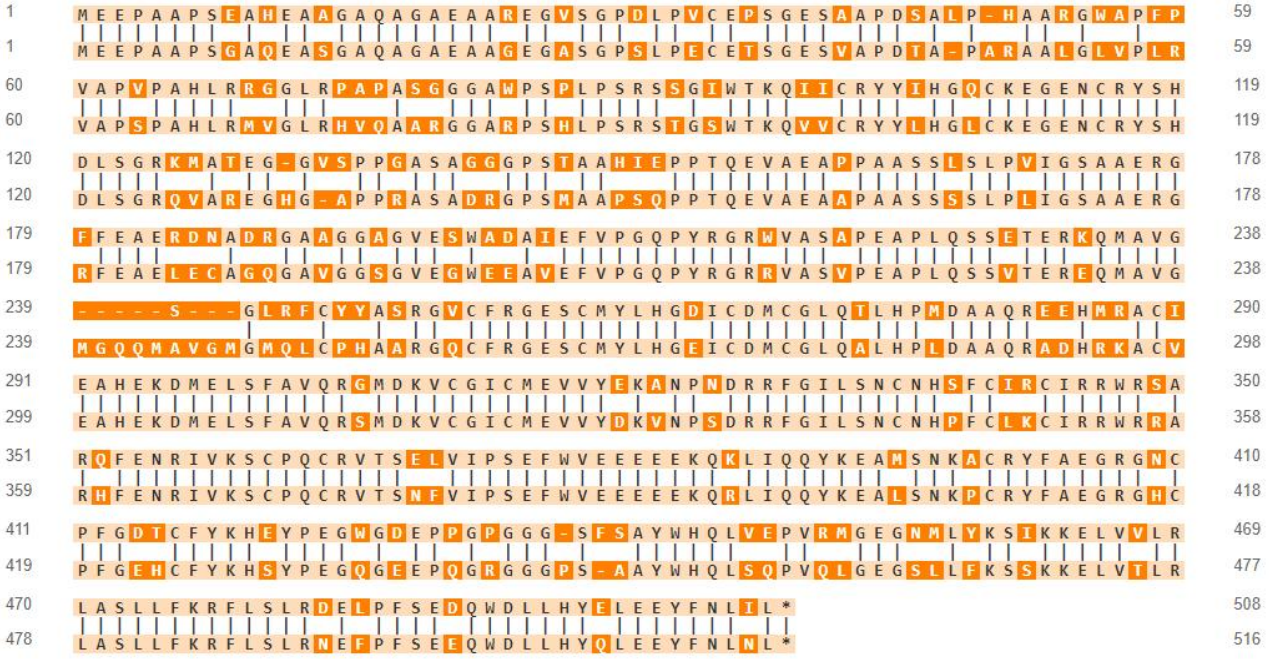

D

```
Matrix: EBLOSUM62
Gap penalty: 2.0
Extend penalty: 2.0
Score: 1691.0
Human length:508
Pig length:499
Alignment length: 529
Identity:      327/529 (61.81%)
Similarity:    374/529 (70.70%)
Gaps:           51/529 (9.64%)
```

Comparison of MKRN3 protein between human and pig

Human vs. Pig: 70.70% similarity

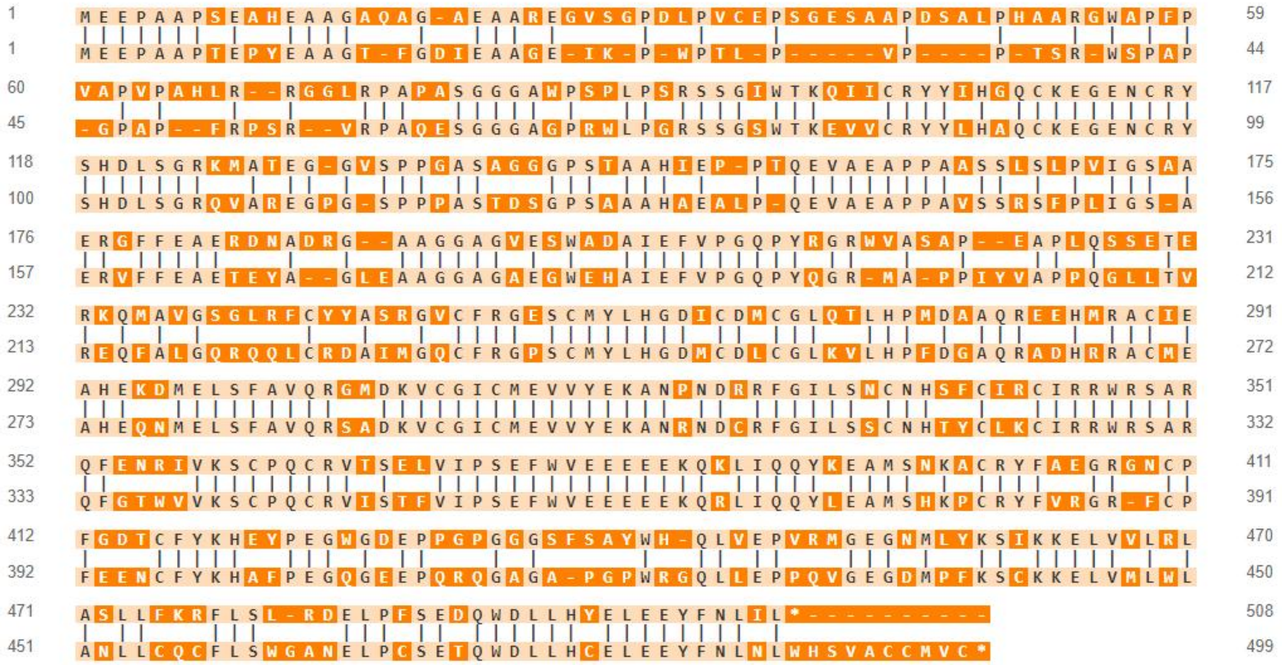

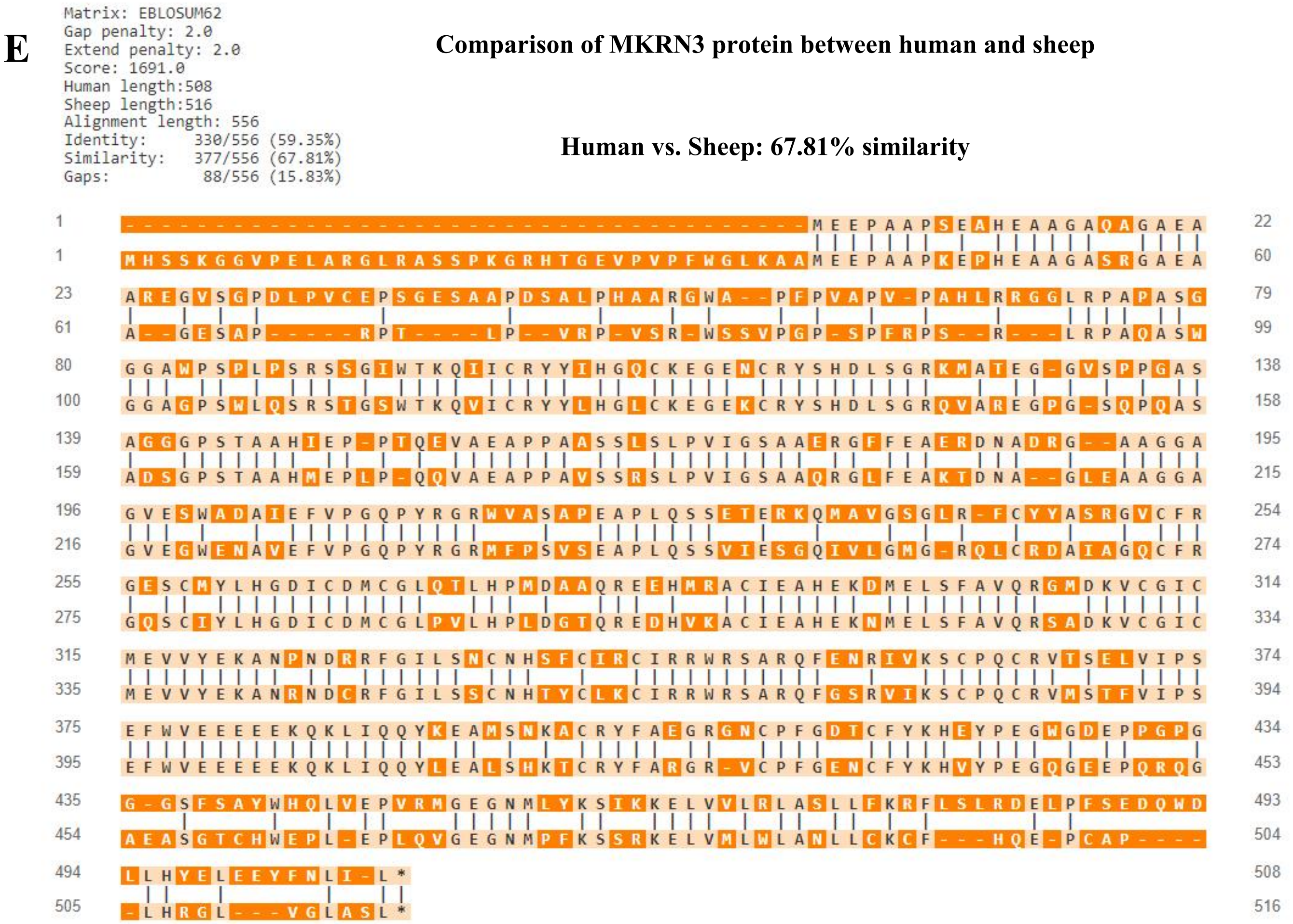
E
Matrix: EBLOSUM62
Gap penalty: 2.0
Extend penalty: 2.0
Score: 1691.0
Human length:508
Sheep length:516
Alignment length: 556
Identity: 330/556 (59.35%)
Similarity: 377/556 (67.81%)
Gaps: 88/556 (15.83%)
Comparison of MKRN3 protein between human and sheep
Human vs. Sheep: 67.81% similarity

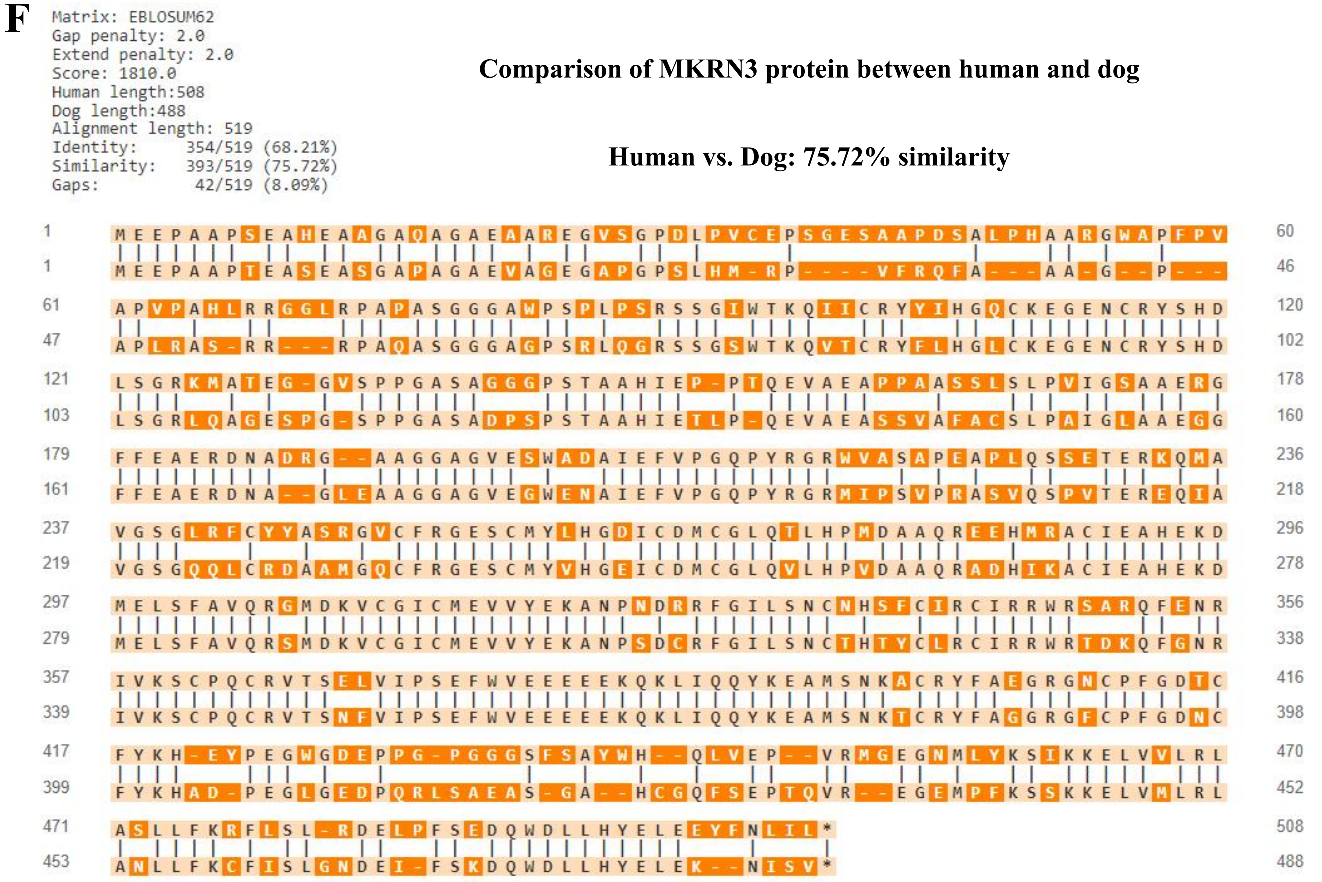
F
Matrix: EBLOSUM62
Gap penalty: 2.0
Extend penalty: 2.0
Score: 1810.0
Human length:508
Dog length:488
Alignment length: 519
Identity: 354/519 (68.21%)
Similarity: 393/519 (75.72%)
Gaps: 42/519 (8.09%)
Comparison of MKRN3 protein between human and dog
Human vs. Dog: 75.72% similarity

G

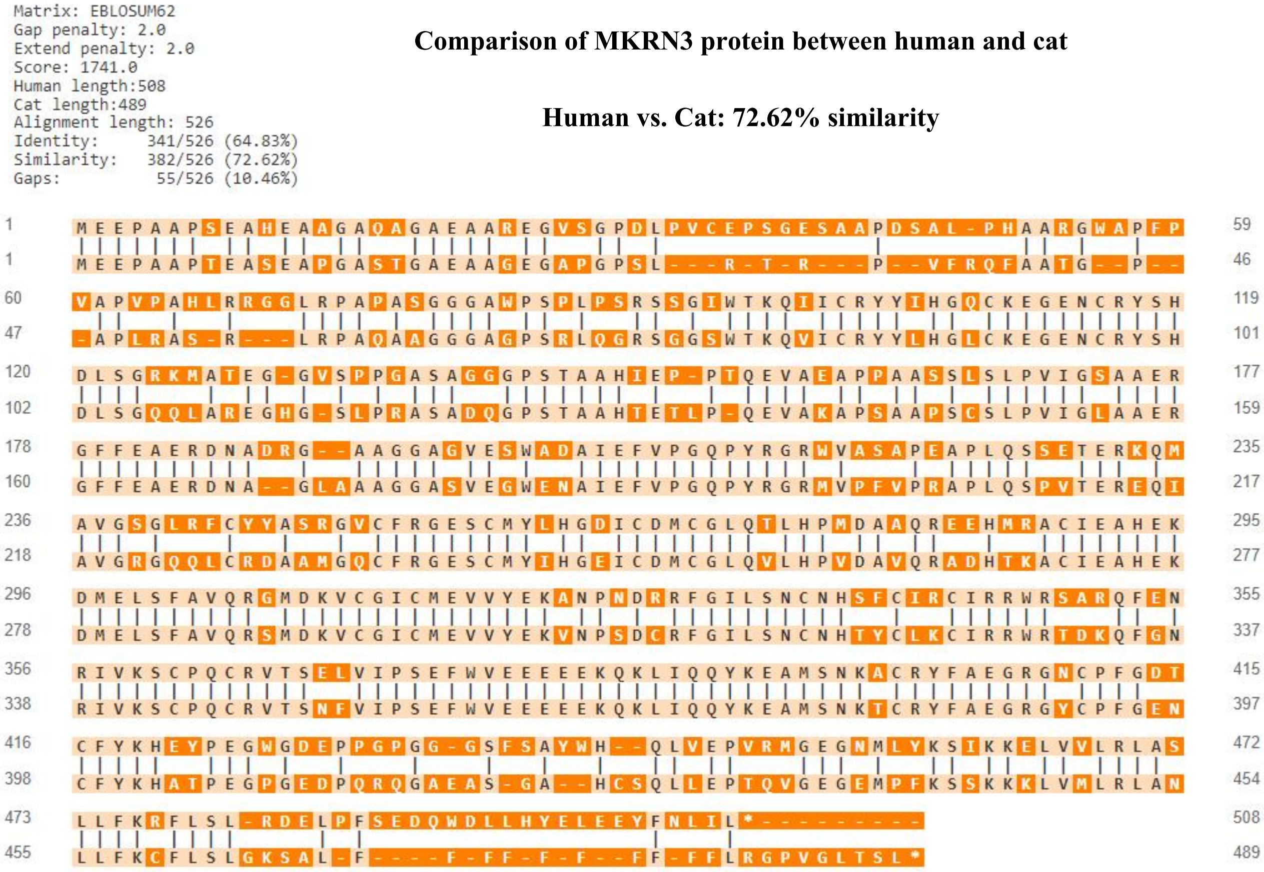

H

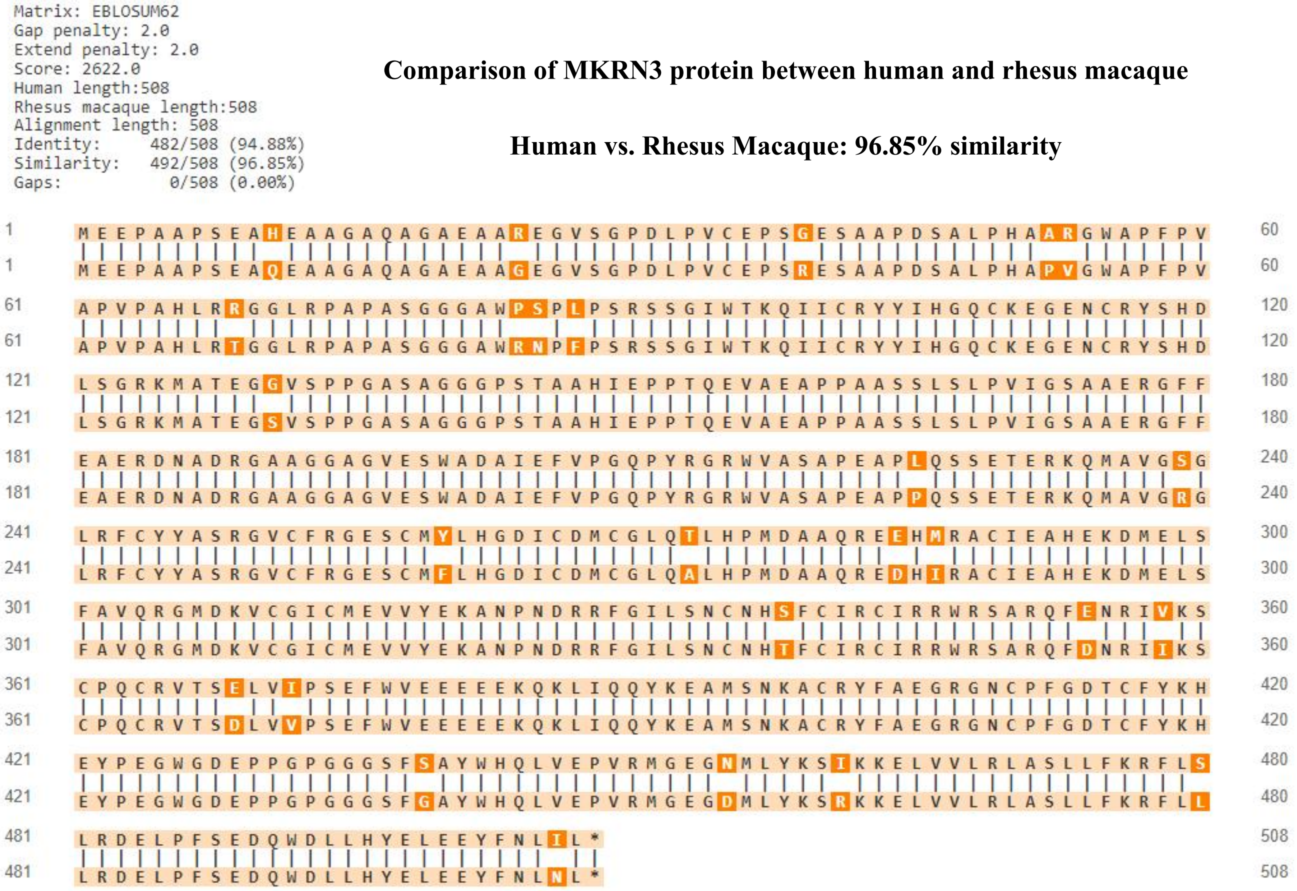

Supplementary Figure 1. Comparison of the MKRN3 protein sequences between humans and various common laboratory animals. (A) Human vs. Mouse: 72.69% similarity; (B) Human vs. Rat: 75.65% similarity; (C) Human vs. Rabbit: 84.20% similarity; (D) Human vs. Pig: 70.70% similarity; (E) Human vs. Sheep: 67.81% similarity; (F) Human vs. Dog: 75.72% similarity; (G) Human vs. Cat: 72.62% similarity; (H) Human vs. Rhesus Macaque: 96.85% similarity.
