# Supplementary material for: A novel model of central precocious puberty disease: Paternal MKRN3 gene–modified rabbit
Source: Animal Model Exp Med. 2025 Jan 24;8(3):511–22. doi: 10.1002/ame2.12544 (PMC11904109; doi:10.1002/ame2.12544)
Supplement: Supplementary file 2 — Figure S2. [file AME2-8-511-s002.pdf]

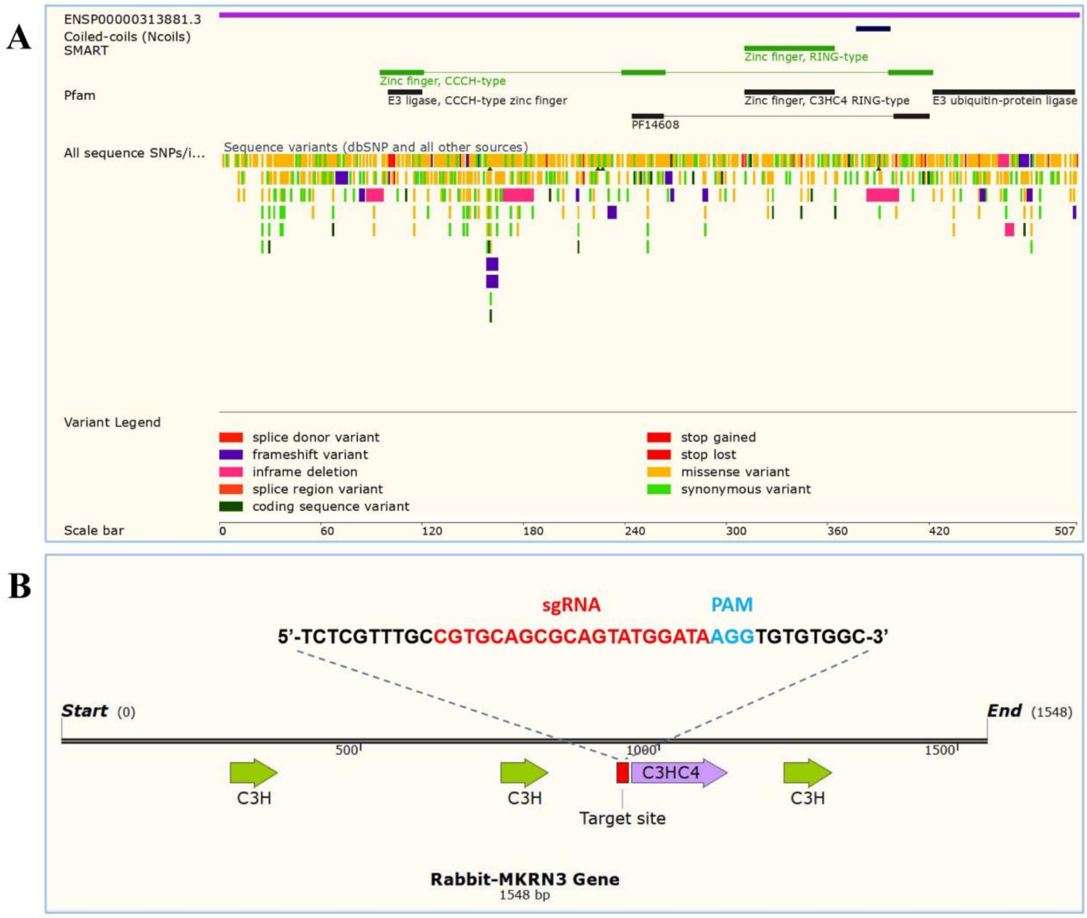

Supplementary Figure 2. Design of sgRNA targeting the C3HC4 domain of rabbit MKRN3. (A) human MKRN3 protein structure and mutation sites (Ensemble, Protein domains for ENSP00000313881.3). (B) SgRNA targeting the C3HC4 domain of rabbit MKRN3.
