# Supplementary material for: A novel model of central precocious puberty disease: Paternal MKRN3 gene–modified rabbit
Source: Animal Model Exp Med. 2025 Jan 24;8(3):511–22. doi: 10.1002/ame2.12544 (PMC11904109; doi:10.1002/ame2.12544)
Supplement: Supplementary file 3 — Figure S3. [file AME2-8-511-s006.pdf]

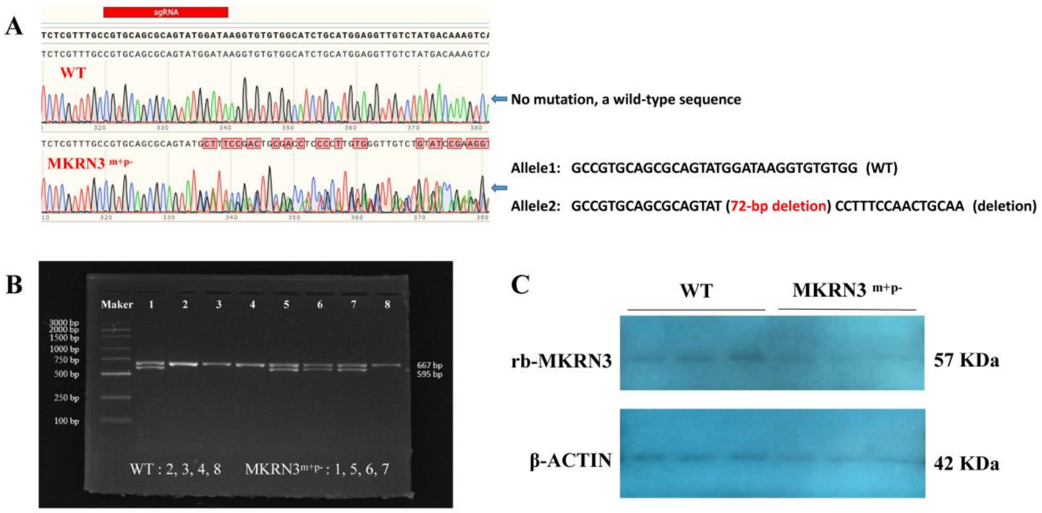

Supplementary Figure 3. MKRN3 mutation rabbits with a deletion of 72 bp. (A) DNA Sanger sequencing results after target site PCR amplification. (B) DNA agarose electrophoresis identification. MKRN3 mutation rabbits with a deletion of 72 bp. (C) The western blot results of MKRN3 mutation rabbits. WT: wild type rabbits; MKRN3$^{m+/p-}$: paternal mutant MKRN3 modified rabbits.
