# Supplementary material for: A novel model of central precocious puberty disease: Paternal MKRN3 gene–modified rabbit
Source: Animal Model Exp Med. 2025 Jan 24;8(3):511–22. doi: 10.1002/ame2.12544 (PMC11904109; doi:10.1002/ame2.12544)
Supplement: Supplementary file 4 — Figure S4. [file AME2-8-511-s009.pdf]

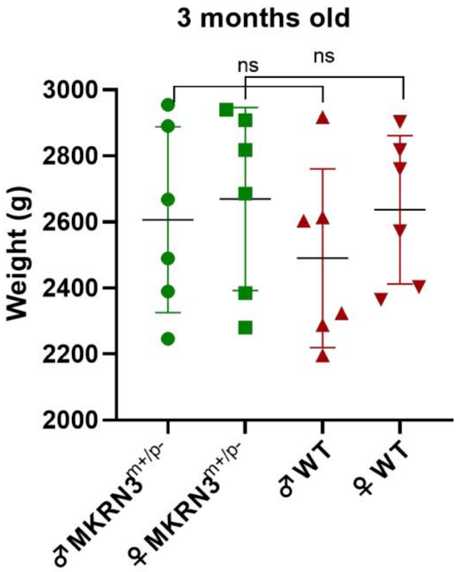

Supplementary Figure 4. The body weight between 3-month-old MKRN3 gene modified rabbits and wild-type rabbits of the same age. There was no significant difference in body weight between 3-month-old MKRN3 gene modified rabbits and wild-type rabbits of the same age and sex (n=6). ns, $p > 0.05$. WT: wild type rabbits; MKRN3$^{m+/p-}$: paternal mutant MKRN3 modified rabbits.
