# Supplementary material for: A novel model of central precocious puberty disease: Paternal MKRN3 gene–modified rabbit
Source: Animal Model Exp Med. 2025 Jan 24;8(3):511–22. doi: 10.1002/ame2.12544 (PMC11904109; doi:10.1002/ame2.12544)
Supplement: Supplementary file 5 — Figure S5. [file AME2-8-511-s004.pdf]

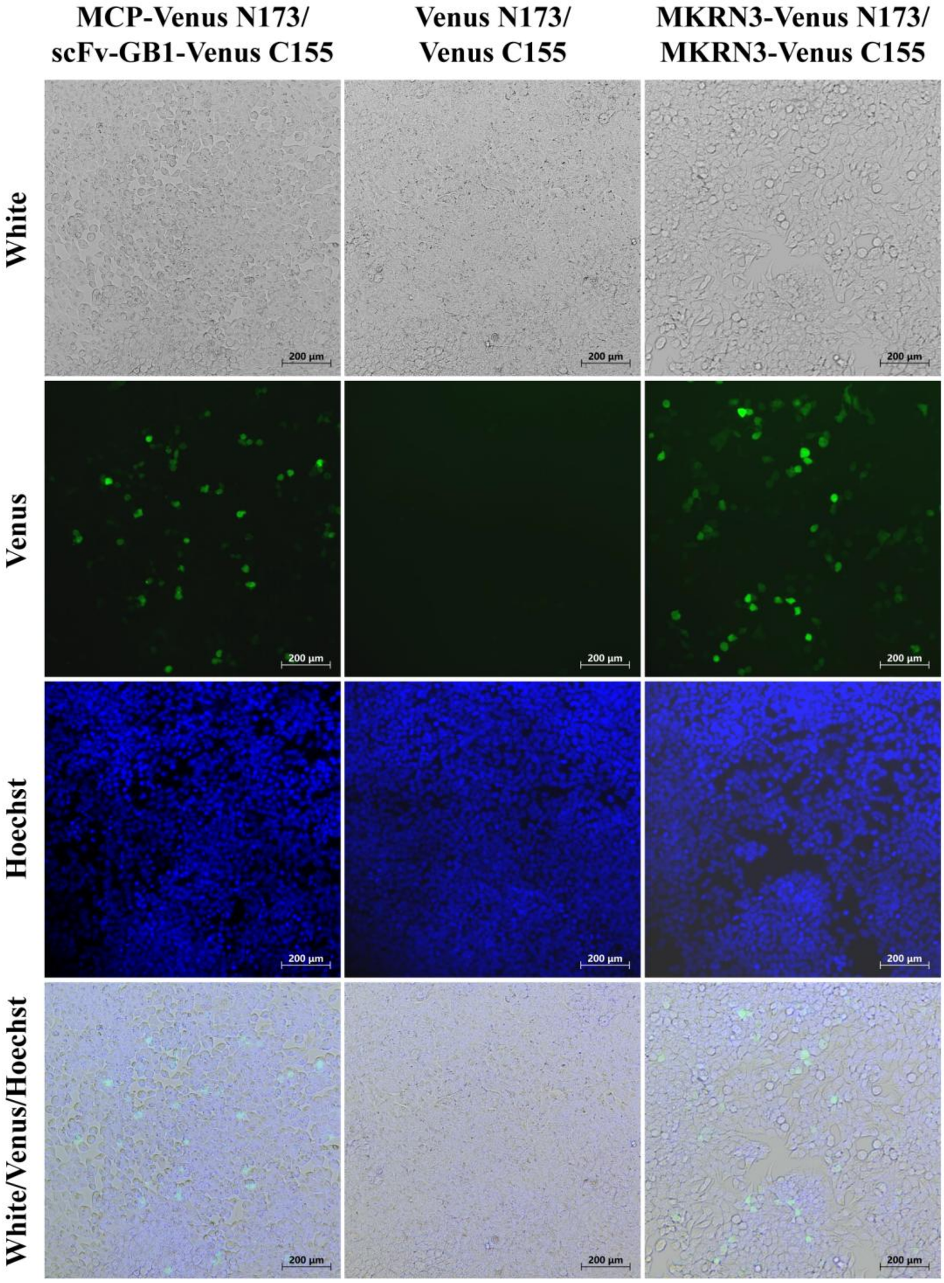

Supplementary Figure 5. rbMKRN3 BiFC result. BiFC experimental results: The result showed significant Venus fluorescence in MKRN3-Venus N173 and MKRN3-Venus C155 transfected into HEK-293T cells, indicating that rbMKRN3 can form homodimers in vitro. NLS-HA-MCP-Venus N173 and scFv-GB1-NLS-Venus C155 are positive controls. Venus N173 and Venus C155 are negative controls. Hoechst : Cell nuclear staining; White: Bright field. Scale bars = 200 μm.
