# Supplementary material for: A novel model of central precocious puberty disease: Paternal MKRN3 gene–modified rabbit
Source: Animal Model Exp Med. 2025 Jan 24;8(3):511–22. doi: 10.1002/ame2.12544 (PMC11904109; doi:10.1002/ame2.12544)
Supplement: Supplementary file 6 — Figure S6. [file AME2-8-511-s008.pdf]

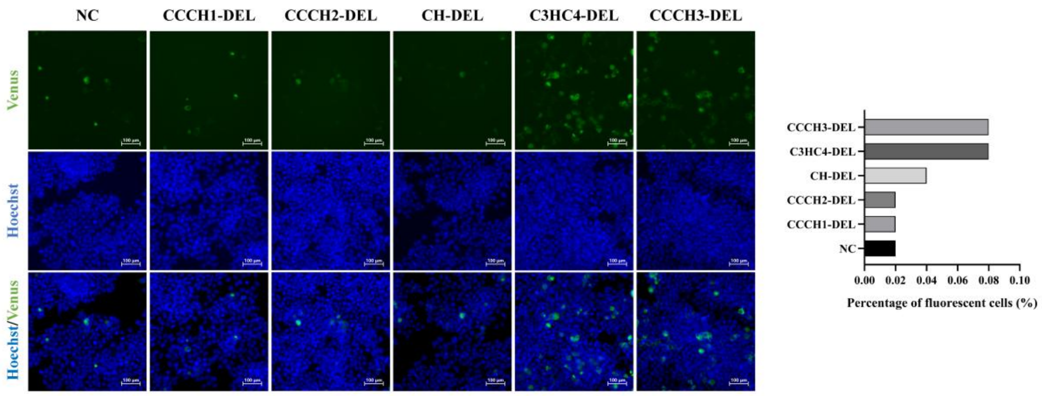

Supplementary Figure 6. BIFC analysis of different MKRN3 mutants. BIFC analysis of different MKRN3 mutants. BiFC plasmid pairs see Supplementary Table 6. NC: MKRN3-Venus N173 and MKRN3-Venus C155; CCCH1-DEL: CCCH1-del-MKRN3-Venus N173 and CCCH1-del-MKRN3-Venus C155; CCCH2 -DEL: CCCH2-del-MKRN3-Venus N173 and CCCH2-del-MKRN3-Venus C155; CCCH3 - DEL: CCCH3-del-MKRN3-Venus N173 and CCCH3-del-MKRN3-Venus C155; CH - DEL: CH-del-MKRN3-Venus N173 and CH-del-MKRN3-Venus C155; C3HC4 - DEL: C3HC4-del-MKRN3-Venus N173 and C3HC4-del-MKRN3-Venus C155. Scale bars = 100 µm. Hoechst: Cell nuclear staining.
