# Supplementary material for: A novel model of central precocious puberty disease: Paternal MKRN3 gene–modified rabbit
Source: Animal Model Exp Med. 2025 Jan 24;8(3):511–22. doi: 10.1002/ame2.12544 (PMC11904109; doi:10.1002/ame2.12544)
Supplement: Supplementary file 7 — Figure S7. [file AME2-8-511-s005.pdf]

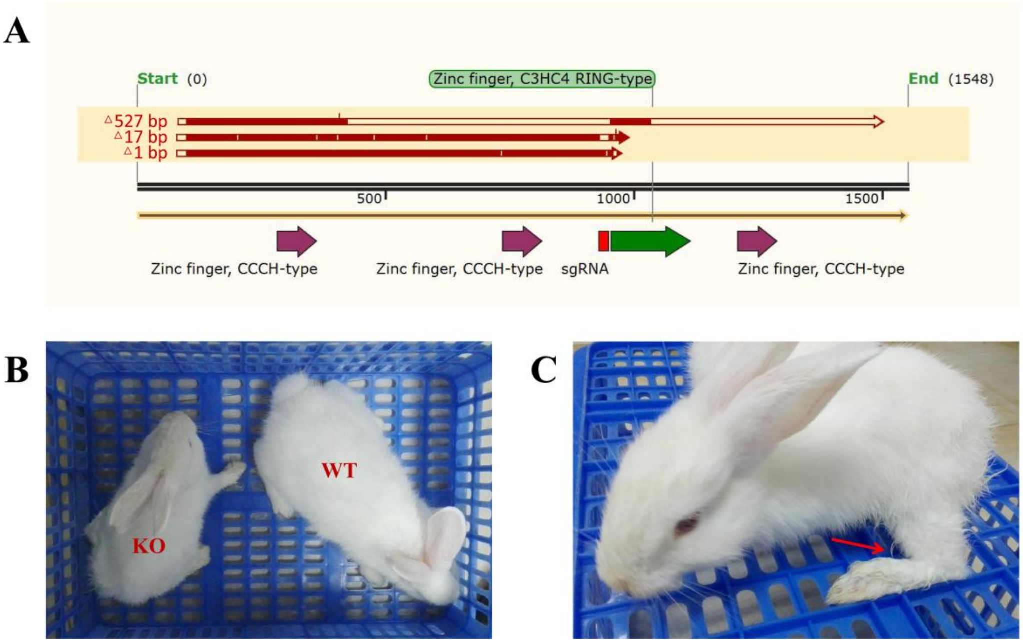

Supplementary Figure 7. Large fragment deletion of MKRN3 complete knockout leads to developmental abnormalities in rabbits. (A) Genotyping analysis revealed that MKRN3 has a large deletion of 527 bp DNA fragments. (B) Complete knockout of large fragments of MKRN3 leads to abnormal development in rabbits. KO: MKRN3 complete knockout rabbits; WT: Wild type rabbits of the same age. (C) The complete knockout of MKRN3 in large fragments gradually resulted in abnormal leg valgus development in rabbits.
