# Supplementary material for: A novel model of central precocious puberty disease: Paternal MKRN3 gene–modified rabbit
Source: Animal Model Exp Med. 2025 Jan 24;8(3):511–22. doi: 10.1002/ame2.12544 (PMC11904109; doi:10.1002/ame2.12544)
Supplement: Supplementary file 8 — Table S1. [file AME2-8-511-s003.pdf]

Supplementary Table 1. Rabbit MKRN3 gene

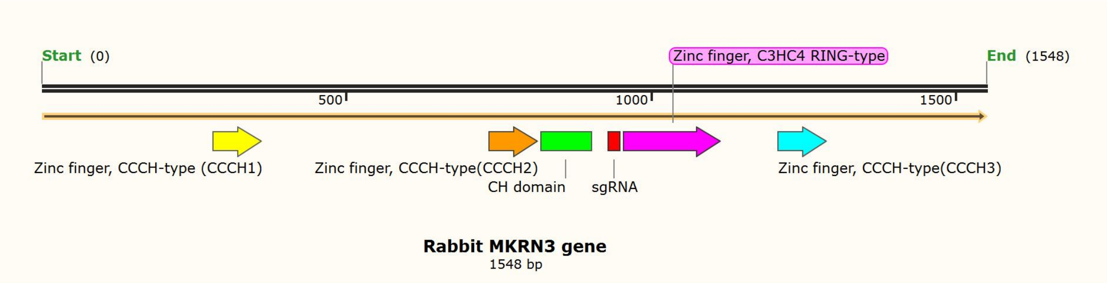

DNA sequence:

5’-ATGGAAGAGCCTGCAGCTCCCTCTGGTGCCCAGGAGGCATCTGGGGCCC

AGGCAGGGGCTGAGGCAGCAGGGGAGGGTGCATCTGGGCCCAGCCTCCCT

GAGTGTGAGACCTCTGGGGAATCTGTGGCTCCAGACACAGCCCCTGCTCG

CGCGGCCTTGGGCCTAGTCCCTCTCCGTGTGGCTCCCAGCCCAGCCCATCT

GCGGATGGTGGGCCTGAGGCACGTCCAGGCCGCAAGGGGAGGGGCCAGG

CCCAGTCACCTGCCGAGCCGGAGCACTGGCAGCTGGACAAAGCAAGTCGT

CTGCAGGTATTATCTGCATGGGCTGTGCAAGGAGGGGGAGAACTGTCGCTA

CTCTCACGACCTTTCTGGCAGGCAGGTGGCCCGAGAGGGCCATGGCGCAC

CGCCCCGGGCCTCTGCAGACAGAGGCCCCAGCATGGCTGCGCCCAGCCAG

CCCCCAACTCAGGAAGTGGCGGAAGCCGCCCCTGCTGCATCCTCAAGCTC

CTTGCCTCTGATTGGCTCGGCTGCTGAAAGGGGTCGCTTCGAAGCCGAGTT

GGAATGCGCTGGTCAAGGGGCTGTCGGAGGATCAGGTGTAGAAGGCTGGG

AAGAGGCCGTTGAGTTTGTTCCCGGGCAGCCCTACCGGGGCCGCAGGGTC

GCTTCTGTCCCCGAGGCTCCTCTACAGAGCTCGGTGACTGAGAGAGAGCA

GATGGCTGTGGGCATGGGGCAGCAGATGGCTGTGGGCATGGGGATGCAAC

TTTGCCCTCACGCTGCCAGGGGACAGTGCTTTCGTGGGGAGAGCTGTATGT

ACCTCCACGGAGAGATATGTGACATGTGTGGGCTACAGGCCTTGCACCCCT

TGGATGCCGCTCAGAGGGCAGACCATAGAAAGGCCTGCGTCGAAGCACAC

GAGAAGGATATGGAGCTCTCGTTTGCCGTGCAGCGCAGTATGGATAAGGTG

TGTGGCATCTGCATGGAGGTTGTCTATGACAAAGTCAACCCCAGCGACCGC

CGCTTTGGCATCCTTTCCAACTGCAACCACCCCTTCTGTCTTAAGTGTATCC

GTAGGTGGAGACGTGCCAGACACTTTGAGAACAGGATCGTCAAGTCCTGC

CCACAGTGCAGAGTCACCTCCAACTTTGTCATTCCCAGTGAGTTCTGGGTG

GAGGAGGAGGAAGAGAAGCAGAGACTTATTCAGCAGTACAAGGAGGCGT

TGAGCAACAAGCCTTGCAGATATTTTGCCGAAGGCAGGGGCCACTGCCCG

TTTGGAGAGCACTGCTTTTACAAGCATTCATACCCTGAGGGCCAGGGAGAG

GAGCCTCAGGGGCGGGGTGGTGGACCGTCGGCCGCATACTGGCATCAACT

TTCGCAGCCTGTGCAGCTGGGAGAGGGCAGCCTGCTCTTTAAAAGCAGTA

AAAAGGAGCTTGTCACGCTTCGGCTGGCCAGTCTGTTGTTTAAGCGGTTTC

TTTCACTGAGAAACGAGTTCCCCTTCTCTGAGGAGCAGTGGGACTTGCTTC

ATTATCAGCTGGAAGAGTATTTCAACTTGAATCTGTAG-3’
