# Supplementary material for: A novel model of central precocious puberty disease: Paternal MKRN3 gene–modified rabbit
Source: Animal Model Exp Med. 2025 Jan 24;8(3):511–22. doi: 10.1002/ame2.12544 (PMC11904109; doi:10.1002/ame2.12544)
Supplement: Supplementary file 9 — Table S2‐S5. [file AME2-8-511-s010.pdf]

Supplementary Table. 2 Oligonucleotides used to obtain sgRNA in vitro transcription DNA templates.

Oligonucleotide	Sequence (5'-3')
RB-MKRN3-crRNA1	AAGCTAATACGACTCACTATAGCGTGCAGCGCAGTATGGAT AGTTTTAGAGCTAGAAATAGCAAG
sgRNAT7common	AAAAGCACCGACTCGGTGCCACTTTTTCAAGTTGATAACGG ACTAGCCTTATTTTAACTTGCTATTTCTAGCTCTAAAAC

Supplementary Table. 3 Primers used for amplification of the MKRN3 target gene loci.

Primers	Sequence (5'-3')
MKRN3-TA-F	GTTGGAATGCGCTGGTCAAG
MKRN3-TA-R	GCTCAACGCCTCCTTGTACT

Supplementary Table. 4 The RT-PCR primers of MKRN3 gene.

Primers	Sequence (5'-3')
MKRN3-RT-PCR-F	GATGCAACTTTGCCCTCAC
MKRN3-RT-PCR-R	TCCTCCACCCAGAACTCACT

Supplementary Table. 5 The primers for qPCR.

Primers	Sequence (5'-3')
GNRH1-qPCR-F	TGACTTTATGCGTGGAAGGCT
GNRH1-qPCR-R	GGCTGATCAACCTCTTTGGC
NPFFR1-qPCR-F	ATCCTCAACCTGGCCGTCA
NPFFR1-qPCR-R	CCAAGCCGCTCATCTTGCAC
LHX8-qPCR-F	ATGGGATTAGTGTGGAAGGC
LHX8-qPCR-R	GTTGTCCTGAGCAAACTGTG
SCGN-qPCR-F	GGAGTTTATGCAGATTTGGCG
SCGN-qPCR-R	TCATGGTGCCTGTGTATTCTG
SHOX2-qPCR-F	TTTTGACGAGACTCACTATCCG
SHOX2-qPCR-R	GAGGACACCTTTATGGAGCTG
GAPDH-qPCR-F	GTATGATTCCACCCACGGCA
GAPDH-qPCR-R	CCAGCATCACCCCACTTGAT
